# Circular mixture model-based characterization of time-of-day seizure patterns during presurgical monitoring

**DOI:** 10.1038/s41598-026-53249-1

**Published:** 2026-05-19

**Authors:** Gonçalo Costa, Matthias Dümpelmann, César Teixeira

**Affiliations:** 1 University of Coimbra, CISUC/LASI – Centre for Informatics and Systems of the University of Coimbra, Coimbra, Portugal; 2 Epilepsy Center, Department of Neurosurgery, Faculty of Medicine, Medical Center-University of Freiburg, University of Freiburg, Freiburg, Germany; 3 Department of Microsystems Engineering—IMTEK, University of Freiburg, Freiburg, Germany

**Keywords:** Clustering, Electroencephalogram, Epilepsy, Presurgical monitoring, Seizure cycles, von Mises, Diseases, Neurology, Neuroscience

## Abstract

Epilepsy is one of the most prevalent neurological diseases. Despite advancements in treatment, Drug-resistant epilepsy continues to pose significant challenges. Identifying temporal patterns in seizure occurrence may provide insight into the mechanisms underlying seizure timing. This study analyzes seizure patterns in patients undergoing presurgical evaluation using the long-term electroencephalogram EPILEPSIAE database. After applying exclusion criteria, 280 patients were analyzed, encompassing 1813 annotated seizure onsets. Seizure timing was treated as a circular variable, and each patient’s data were fit to a von Mises Mixture Model, with each component representing a cluster of seizure onset times. Clustering strength was evaluated using the Bayesian Information Criterion by comparing each model against a uniform one. To address the increased false positive rate associated with small sample sizes, primary results are reported for patients with more than 8 seizures. In this subgroup, model-supported evidence of time-of-day seizure onset clustering was found in 30% of patients. Results from patients with fewer seizures are reported separately as exploratory analyses due to reduced model stability at low sample sizes. These findings demonstrate that, for some patients, structured time-of-day patterns can be detected even during presurgical monitoring, where medication tapering, sleep disruption, and the hospital environment may perturb seizure timing, suggesting that rhythmic influences on seizure occurrence may persist despite substantial environmental perturbations.

**Supplementary Information:**

The online version contains supplementary material available at 10.1038/s41598-026-53249-1.

## Introduction

A significant concern for individuals with epilepsy is Drug-resistant epilepsy (DRE), affecting nearly one-third of patients. In such cases, Anti-Seizure Medication (ASM) fail to provide adequate seizure control, preventing patients from leading seizure-free lives^1,2^. When seizure control cannot be achieved, the objective becomes providing the patient with relevant and trustworthy information about their timing, thereby substantially reducing the disease’s burden^3^.

For hundreds of years, researchers have been interested in studying the possible periodicity of seizure occurrence. Reports suggesting seizures might not be entirely randomly generated date back to the 18th century: Mead and Stack (1748)^4^ studied the effect of lunar changes on different diseases, highlighting the occurrence of “some fits” on “every new and full moon.” In the late 19th century, more experimental and objective studies surrounding epilepsy arose. In 1881, Gowers^5^ studied the day/night effect in 850 patients; in 1929, Langdon-Down and Brain^6^ reported time-of-day (TOD) peaks of seizure occurrence in 66 patients; in 1938, Griffiths and Fox^7^ performed a patient-specific analysis of 114 patients and found cases of circadian and multidien rhythms. Over the last 15 years, the development of devices for long-term and ultra-long-term continuous recording of seizure activity, namely the Electroencephalogram (EEG), has brought significant advances in seizure prediction, forecasting, and the exploration of periodicities.

Presurgical monitoring provided the opportunity to record patients’ EEGs over several days. Multiple databases have been created using the long-term EEG recorded in this context, such as EPILEPSIAE^8^ and CHB-MIT^9^. During presurgical monitoring, to identify the epileptogenic zone, patients must have seizures. To this end, patients are subject to ASM withdrawal and sleep deprivation. Furthermore, in most cases, these patients are restricted to their beds and constantly observed, leading to higher levels of stress and anxiety^10–12^. These conditions create brain dynamics that do not align with patients’ day-to-day lives.

A significant breakthrough came with advances in ambulatory continuous EEG recordings, allowing the creation of longer databases such as NeuroVista^13^ and NeuroPace^14^. The ability to record several months or years of continuous EEG data allows for a better understanding of longer cycles of seizure activity, considered informative for seizure forecasting models. Several studies have already identified circadian or multidien cycles of seizure occurrence by using this ultra-long-term data to explore cyclic patterns^15–20^. Consequently, the focus shifted away from presurgical monitoring datasets. As a result, there is a lack of studies exploring seizure cycles within presurgical settings. This is particularly true for the EPILEPSIAE database, which remains one of the most extensive datasets available for presurgical patients. However, presurgical monitoring provides a complementary perspective on seizure timing compared with ultra-long-term ambulatory recordings. In this setting, seizures are intentionally facilitated through ASM tapering and sleep disruption, while patients are exposed to a highly structured hospital environment. These factors represent strong perturbations that could plausibly obscure TOD seizure organization. Examining whether non-uniform seizure timing remains detectable under such conditions offers insights into seizure timing in presurgical populations and serves as a stress test for the robustness of TOD structure in seizure occurrence. Furthermore, presurgical datasets offer a high seizure occurrence over relatively short recording periods, allowing patient-level analyses of daily seizure timing without the need for prolonged monitoring. While such recordings cannot replace ultra-long-term studies aimed at characterizing multi-day cycles or long-term stability, they may provide an efficient setting to assess whether TOD clustering is present at all and to inform further analyses in datasets with longer observation periods. Accordingly, we developed an approach to characterize these TOD clusters of seizure onsets during presurgical monitoring. Given that seizure timings are inherently circular within the 24-hour day, we modeled their distribution using finite mixtures of von Mises models, allowing for the identification of multiple preferred diurnal phases of seizure occurrence. This framework enables the detection and quantification of multimodal TOD clustering and distinguishes structured daily seizure timing from random occurrence.

## Materials and methods

After visualizing seizure onset times across patients, we found several cases in which the onset times clustered around specific TODs. This experiment aims to develop a quantitative method to identify these patients, model the seizure occurrence behavior, and differentiate them from others with randomly occurring seizures throughout the day.

**Fig. 1:**
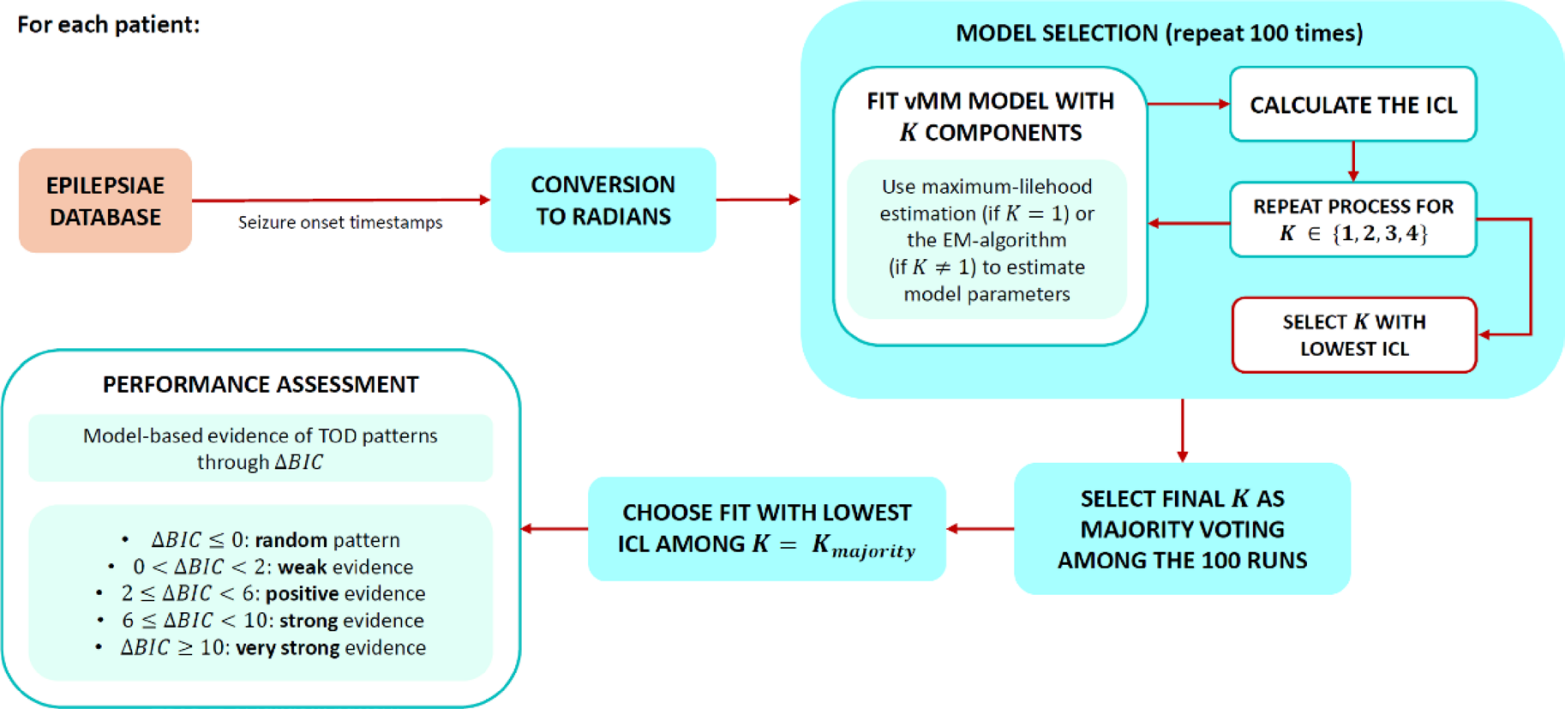
Overview of the proposed von Mises Mixture Model-based seizure onset TOD pattern identification pipeline. vMM stands for von Mises Mixture model, ICL for Integrated Complete Likelihood, and BIC for Bayesian Information Criterion.

To reduce potential bias from closely spaced seizures within clusters, only lead seizures separated by at least 4 h were considered. The choice of a 4-hour lead seizure separation criteria was guided by previous work^21–28^. Additionally, seizures with missing onset times or patients with fewer than three lead seizures were excluded to ensure data reliability. Figure 1 presents an overview of the employed von Mises Mixture Model-based seizure onset TOD pattern identification pipeline.

### Data

The data source for the present study is the EPILEPSIAE^8^ database. This database contains long-term EEG recordings and metadata from patients with epilepsy collected during presurgical monitoring. There are over 2000 days of recordings, amounting to nearly 50,000 h. Data were recorded at the epilepsy centers of the Hôpital de la Pitié-Salpêtrière (HdlPS) in Paris, France; the University Hospital of Coimbra (HUC), Portugal; and the University Hospital Freiburg (UKLFR), Germany. The Ethical Committees of the three hospitals involved in the EPILEPSIAE database approved the use of this data for research purposes (Ethik-Kommission der Albert-Ludwigs-Universität, Freiburg; Comité consultatif sur le traitement de l’information en matière de recherche dans le domaine de la santé, Pitié- Salpêtrière University Hospital; and Ethics Committee of the Centro Hospitalar e Universitário de Coimbra). All studies were carried out in accordance with the necessary guidelines and regulations, and all subjects and/or their legal guardian(s) provided informed written consent.

There is data from 285 patients (53% male and 47% female, aged 2 to 67 years, with a mean age of 34.30 years) with a total of 2860 reported seizures (ranging from 2 to 94, with a mean of 10 seizures). Seizure events were annotated by experienced clinical staff based on video analysis and EEG screening, including electrographic and clinical seizure onset and offset times. The EEG was acquired from scalp electrodes in 219 patients (77%) and from iEEG in 66 patients, using stereotactically implanted depth electrodes, subdural grids, and/or strips. Some iEEG patients also have scalp EEG recordings, either having undergone each in separate hospital admissions or having both recorded simultaneously. Seizures originated exclusively in the temporal lobe in 170 patients (60%) and in the frontal lobe in 24 patients (8%). Occipital and parietal lobe seizure onset was reported in 3 patients each. Regarding lateralization, seizures originated from the left hemisphere in 86 patients (30%), from the right hemisphere in 79 patients (28%), and bilaterally in 57 patients (20%). Some patients had seizures originating from multiple lobes, and localization or lateralization information was not available for all patients. More detailed patient-level information is provided in Table S1 of the Supplementary Materials.

### Preprocessing

To search for patterns around the day, we look at the day as a circle and the lead seizure onset TOD as a circular variable. To this end, we converted each TOD to the number of minutes since midnight and subsequently mapped it to a corresponding radian value between -π and π:

1 $$\:\theta\:=2\pi\:\frac{t}{1440}-\pi\:\:,$$

where *\:t* corresponds to the onset in minutes since midnight and $\:\theta\:\in\:[-\pi\:,\pi\:)$ to the seizure onset mapped in radians. All seizure onset times were analyzed using local clock time at each recording center. Daylight-saving time transitions were not explicitly corrected for, as presurgical monitoring periods are short (panning only a few consecutive days), making any shifts unlikely to meaningfully affect the within-admission TOD analyses.

### Model structure

To describe these patient-specific TOD patterns of lead seizure onset times, we model the circular distribution of seizure onset phases using a finite mixture of von Mises distributions. This framework can capture both unimodal and multimodal clusters of lead seizure onset times, accounting for the circular nature of TOD data. Each mixture model’s parameters were estimated using maximum likelihood through an Expectation–Maximization (EM) algorithm. All implementations of the von Mises distributions and the mixture models were obtained using the *vonMisesMixture*^29^ Python package.

#### The von Mises distribution

The von Mises probability distribution describes unimodal data on the unit circle and provides the building block of the proposed model. This distribution is the standard analogue for the Gaussian distribution on circular data^30,31^. The von Mises distribution is a special case of the von Mises-Fisher^32^ distribution for univariate variables and its probability density function given by:

2 $$\:{f}_{vM}\left(\theta\:\right|\mu\:,\kappa\:)=\frac{{e}^{\kappa\:\mathrm{c}\mathrm{o}\mathrm{s}(\theta\:-\mu\:)}}{2\pi\:{I}_{0}\left(\kappa\:\right)},$$

where $\:\theta\:\in\:[-\pi\:,\pi\:)$ corresponds to the onset phase. $\:\mu\:$ and $\:\kappa\:$ correspond to the parameters of the von Mises distribution: the direction/center and the width/concentration, respectively. Large $\:\kappa\:$ values correspond to concentrated clusters around $\:\mu\:$, and the distribution approaches uniformity as $\:\kappa\:$ approaches zero. $\:{I}_{0}\left(\kappa\:\right)$ represents the zero-th order Bessel function of first kind^33–37^.

The parameters of one von Mises model are obtained by direct maximum-likelihood estimation^35^. For more information on the implementation of the maximum-likelihood estimation framework, please refer to Sect. 2 of the supplementary materials.

#### The von Mises mixture model

Given that many patients exhibited seizures clustered around multiple preferred TODs, we extended the single-component von Mises model to a finite mixture of von Mises distributions. This approach allows several peaks on the 24-hour circle while maintaining a continuous, periodic probability density.

Considering the seizure onset phases $\:\theta\:\in\:[-\pi\:,\pi\:)$, the von Mises Mixture Model (vMM) is defined as:

3 $$\:{f}_{vMM}\left(\theta\:\right|\omega\:,\mu\:,\kappa\:)=\sum\:_{k=1}^{K}{\omega\:}_{k}{f}_{vM}\left(\theta\:|{\mu\:}_{k},{\kappa\:}_{k}\right)\:,$$

where *\:K* is the number of mixture components, $\:{\omega\:}_{k}$ is the mixing weight, a nonnegative quantity such that $\:\sum\:_{k=1}^{K}{\omega\:}_{k}=1$, $\:{f}_{vM}$ is a single-component von Mises model with mean direction $\:{\mu\:}_{k}$ and concentration $\:{\kappa\:}_{k}$, and $\:\omega\:=({\omega\:}_{1},...,{\omega\:}_{K})$, $\:\mu\:=({\mu\:}_{1},...,{\mu\:}_{K})$, and $\:\kappa\:=({\kappa\:}_{1},...,{\kappa\:}_{K})$ are the vectors of mixture weights, component mean directions, and component concentrations, respectively^33,34,36–42^.

The mixture parameters $\:{\mu\:}_{k}$, $\:{\kappa\:}_{k}$, and $\:{\omega\:}_{k}$ were estimated by maximum likelihood using the Expectation-Maximization (EM) algorithm. For more information on the implementation of the EM algorithm, please refer to Sect. 3 of the supplementary materials.

### Model selection

After estimating mixture parameters for each number of components *\:K*, model selection aimed at identifying the number of clusters with the best trade-off between model simplicity, goodness-of-fit, and cluster separation.

For each patient, we fit and compared vMMs with one to four components. Many patients in presurgical monitoring have a small number of lead seizures, and finite-mixture models become difficult to identify and highly unstable when the number of samples per component becomes too small^38,42,43^. For this reason, we considered a maximum of 4 clusters per day. Limiting the number of components stabilizes the EM estimation, avoids overfitting, and ensures reliable model selection.

Then, we used the Integrated Completed Likelihood (ICL) criterion to select the model with the best-defined clusters. The ICL is widely used for finite mixture model-based clustering problems, as it penalizes overlapping components^43–47^.

#### The Integrated Complete Likelihood criterion

Considering a vMM fitted to *\:n* seizure onset phases $\:{\theta\:}_{1},...,{\theta\:}_{n}\in\:[-\pi\:,\pi\:)$, the maximized log likelihood is:

4 $$\log \hat{L} = \mathop \sum \limits_{{i = 1}}^{n} \log ~\left( {\mathop \sum \limits_{{k = 1}}^{K} \hat{\omega }_{k} f_{{vM}} \left( {\theta _{i} {\mid }\hat{\mu }_{k} ,\hat{\kappa }_{k} } \right)} \right).$$

where $\:\hat {{\omega\:}}_{k}$ is the mixing weight, and $\:{f}_{\mathrm{v}\mathrm{M}}$ is the density of a single-component von Mises model with estimated mean direction $\:\hat {{\mu\:}}_{k}$ and concentration $\:\hat {{\kappa\:}}_{k}$.

The Bayesian Information Criterion (BIC) is given by:

5 $$BIC = - 2\log \left( {\hat{L}} \right) + p~\log \left( n \right),$$

where *\:p* is the number of free parameters. The total number of free parameters is $\:p\:=\:3K-1$, accounting for *\:K* mean directions, *\:K* concentration parameters, and *\:K-1* independent mixture weights (the last weight being determined by the unit-sum constraint)^43,48,49^.

The first term of the BIC, $- 2\log \left( {\hat{L}} \right)$, rewards the goodness-of-fit, while the second term, $\:p\:log\left(n\right)$, penalizes complexity to avoid over-fitting, as it increases with a larger number of free parameters *\:p*. However, the BIC does not consider how well the mixture components are separated. Accordingly, the ICL adds a classification-entropy term to further penalize overlapping or poorly defined clusters:

6 $$\:ICL=BIC+2H\:.$$

Considering the posterior probabilities (responsibilities) $\:{\tau\:}_{ik}$ defined in Eq. 3 of Sect. 3 of the supplementary materials, the classification entropy is given by:

7 $$\:H=-\sum\:_{i=1}^{n}\:\sum\:_{k=1}^{K}{\tau\:}_{ik}\mathrm{log}({\tau\:}_{ik})\:.$$

Lower ICL values indicate models capable of fitting the data, without adding too much complexity and maintaining well-separated clusters^42,43,46,50^.

#### Model selection protocol

Due to the relatively small number of samples on some patients, along with the stochastic nature of the EM algorithm (randomly initialized variables), the fit and model selection of the vMMs were at times unstable. To tackle this problem, the selection of the best number of clusters (mixture components) was repeated 100 times with different random initializations.

Within each restart, each patient’s seizure onset phases $\:\theta\:\in\:\left[-\pi\:,\pi\:\right)$ were fit for $\:K\in\:\left\{\mathrm{1,2},\mathrm{3,4}\right\}$ mixture components. Then, the ICL was calculated for each model, and the number of components *\:K* of the model producing the lowest ICL was chosen. Across the 100 runs, the most frequently selected number of components, $\:{K}_{majority}$, was identified, and among the models with $\:K={K}_{majority}$, the one with the lowest ICL was chosen as the final solution.

In conclusion, the final patient-specific model corresponds to the vMM configuration with $\:{K}_{majority}$ components, $\:\mu\:$ mean directions, $\:\kappa\:$ concentrations, and $\:\omega\:$ mixture weights, which produced the smallest ICL among the solutions with $\:K={K}_{majority}$. This majority-vote consensus ensures robustness of the selected model to initialization variability while preserving reproducibility across runs.

### Performance assessment

After model selection, we wanted to evaluate the strength of evidence that seizure onsets were clustered around specific TODs rather than uniformly distributed across the day for each patient. To this end, we used the difference of Bayesian Information Criterions ($\:\varDelta\:BIC$) to compare the selected mixture model to a reference uniform model.

The $\:\varDelta\:BIC$ derives its significance from Bayesian statistics, more specifically, the Bayes Factor (BF). The BF, calculated as:

8 $$\:B{F}_{21}=\frac{p\left(data|{H}_{2}\right)}{p\left(data|{H}_{1}\right)}\:,$$

is a ratio of marginal likelihoods which expresses how much more the data supports model $\:{H}_{2}$ over $\:{H}_{1}$. The $\:{\Delta\:}BIC$ provides an approximation to the BF, such that:

9 $$\:2log\left(B{F}_{21}\right)\approx\:{\Delta\:}BIC=BI{C}_{1}-BI{C}_{2}\:.$$

To illustrate this in practice, consider the following example: if $\:{\Delta\:}BIC\:=\:BI{C}_{1}\:-\:BI{C}_{2}\:=\:10$, then model $\:{H}_{2}$ explains the data $\:{e}^{\frac{10}{2}}\approx\:150$ times better than model $\:{H}_{1}$^48,51^.

For guidelines on the interpretation of different $\:{\Delta\:}BIC$ values, we refer to Kass and Raftery (1995)^48^. For $\:{\Delta\:}BIC$ values: less than 0, there is no evidence against $\:{H}_{1}$ (random); from 0 to 2, the evidence against $\:{H}_{1}$ is “not worth more than a bare mention” (weak); from 2 to 6, there is “positive” evidence against $\:{H}_{1}$; from 6 to 10 there is “strong” evidence against $\:{H}_{1}$; greater than 10, there is “very strong” evidence against $\:{H}_{1}$.

Accordingly, in this study, the $\:{\Delta\:}BIC$ is calculated as:

10 $$\:{\Delta\:}BIC=BI{C}_{uniform}-BI{C}_{vMM}\:,$$

where $\:BI{C}_{uniform}=2nlog\left(2\pi\:\right)$, as we consider that $\:{p}_{uniform}\left(\theta\:\right)=\frac{1}{2\pi\:}$ for every seizure onset phase $\:\theta\:\in\:[-\pi\:,\pi\:)$ and that the uniform model has no parameters to estimate.

Finally, following Kass and Raftery (1995), we define a patient as having statistical evidence for seizure onset TOD patterns if the fitted von Mises mixture model (clustered) yields “positive”, “strong” or “very strong” Bayesian evidence relative to the uniform model (random), i.e., if $\:{\Delta\:}BIC\:>\:2$. Under this criterion, $\:{\Delta\:}BIC$ values between 2 and 6 correspond to “positive” evidence (BF approximately from 3 to 20), values between 6 and 10 to “strong” evidence (BF approximately from 20 to 150), and values greater than 10 to “very strong” evidence (BF approximately over 150). Patients with negative $\:{\Delta\:}BIC\:$are considered to show insufficient evidence to reject uniform (random) seizure timing, while $\:{\Delta\:}BIC$ values from 0 to 2 correspond to “weak” evidence, which is evidence of negligible strength. Furthermore, when dealing with small sample sizes, cases where the selected model as the same number of components *K* as lead seizures (only singleton clusters) are classified as “random”.

### Statistical analysis

In order to understand the effect of sample size on the tendency of the vMM pipeline to detect TOD seizure onset clustering, we estimated the FPR for different numbers of lead seizures. Firstly, for each number of seizures *n* for which we intend to estimate the FPR, we generated *n* random samples of circular seizure onset phases. Then, we applied the methodology previously described to the randomly generated data. Finally, we repeated this process 200 times and recorded the percentage of runs in which the random data is classified as “clustered” for each number of seizures *n*. This analysis quantifies how strongly the classification depends on the number of available lead seizures per patient.

To assess whether the prevalence of TOD clustering differed across subgroups of patients, we compared the proportion of patients classified as “clustered” versus “random” using contingency-table analyses. Patients classified as having weak evidence were not included in these comparisons. Given the small number of patients in several subgroups, we applied Fisher’s exact test. For contingency tables larger than 2 × 2, we used the Fisher–Freeman–Halton extension of Fisher’s exact test. To account for the multiple subgroup comparisons performed in Table 2, p-values were adjusted using the Holm-Bonferroni method. Statistical significance was assessed considering a 5% significance level (α = 0.05).

## Results

This study was based on seizure onset metadata from the EPILEPSIAE^8^ database. This database comprises 285 patients with a total of 2860 recorded seizures. Of the 2860 seizures, four had no seizure onset information, so they were discarded. Furthermore, to account for seizure clusters, we set a 4-hour separation criterion between lead seizures. Therefore, of the 2856 remaining seizures, 1822 seizure onset times were left. Finally, due to the lead seizure criterion, five patients had fewer than three lead seizures, so they were discarded. The final dataset comprised 1813 lead seizure onset times from 280 patients.

**Fig. 2:**
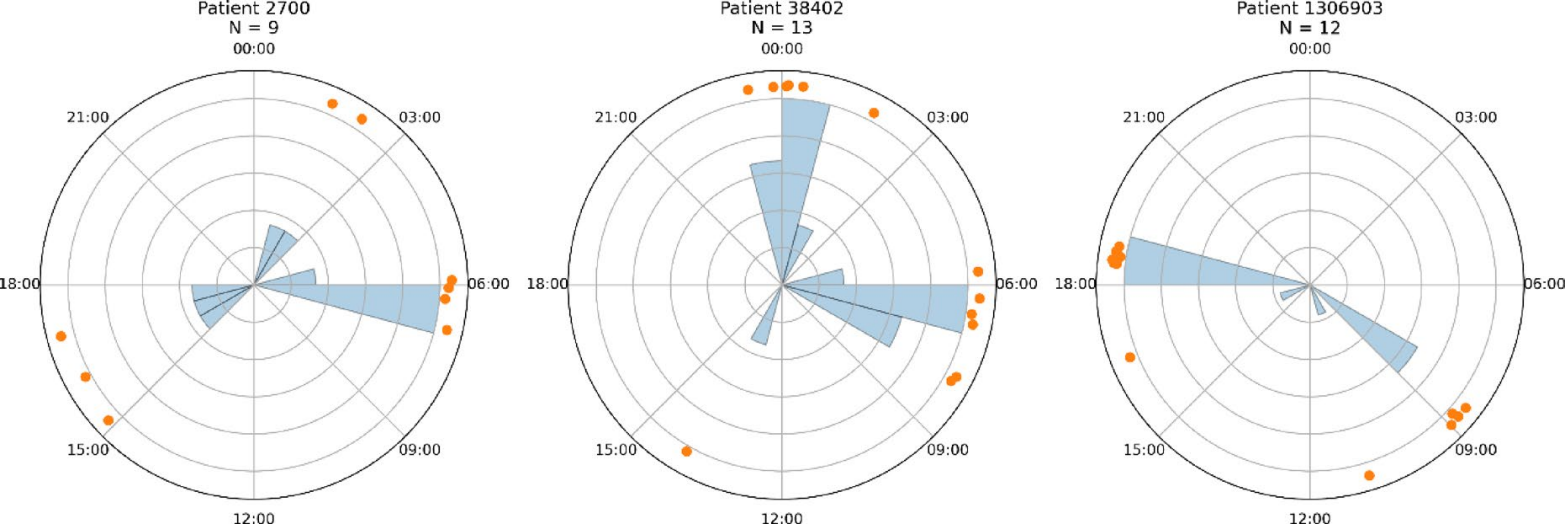
Lead seizure onset times throughout the day for three patients who appear to have most of their seizures centered around specific times during the day.

Figure 2 shows three examples of patients with seemingly significant TOD seizure occurrence patterns. For each patient, the lead seizure onset times appear to be clustered around specific TODs.

These are the types of patient-specific TOD patterns we aim to identify with this study. However, the number of lead seizures available per patient is a critical factor for interpreting the vMM classifications. Therefore, this section first assesses how seizure count affected the FPR of the proposed pipeline. Based on this analysis, patients with more stable model evidence are then first analyzed as the primary cohort, while the remaining patients are reported separately as an exploratory analysis, in order to provide a complete overview of the dataset while avoiding overinterpretation of classifications obtained from sparse data.

### False positive analysis

A major concern in this study is the limited number of lead seizures available for many patients. As our goal is to study patient-specific patterns, the analysis is limited to the number of lead seizures available for each patient. This is particularly relevant because mixture models can overfit small sample sizes and over-identify apparent structure. Therefore, before interpreting the patient-specific vMM results, we assessed the effect of small sample sizes on the model’s tendency to classify randomly generated seizure times as clustered.

To this end, we used the FPR strategy described in the Methods section. Briefly, for each number of seizures *\:n*, we generated random circular seizure onset phases from a uniform distribution, applied the full vMM pipeline, and recorded the proportion of simulations classified as “clustered”. Table 1 presents the FPR for sample sizes from 3 to 10.

**Table 1:** FPR values for sample sizes of 3 to 10 seizures. FPR stands for false positive rate.

*n*	3	4	5	6	7	8	9	10
FPR	0.78	0.66	0.53	0.36	0.25	0.19	0.11	0.07

As shown in Table 1, the FPR was strongly dependent on the number of lead seizures. For patients with fewer than 9 lead seizures, the FPR exceeded 11%, indicating reduced reliability of the vMM clustering classification in this range. This means that, under sparse-data conditions, model-supported clustering may partly reflect the limited number of observations rather than a stable patient-specific TOD pattern.

Accordingly, to ensure more robust and interpretable results, the main analyses reported below focus on patients with at least 9 lead seizures, for whom the model showed greater stability. This subset contains 50 patients with a total of 600 lead seizures. Results for patients with fewer than 9 lead seizures are presented separately as an exploratory analysis to provide a complete overview of the dataset.

### Primary analysis: patients with ≥ 9 seizures

**Fig. 3:**
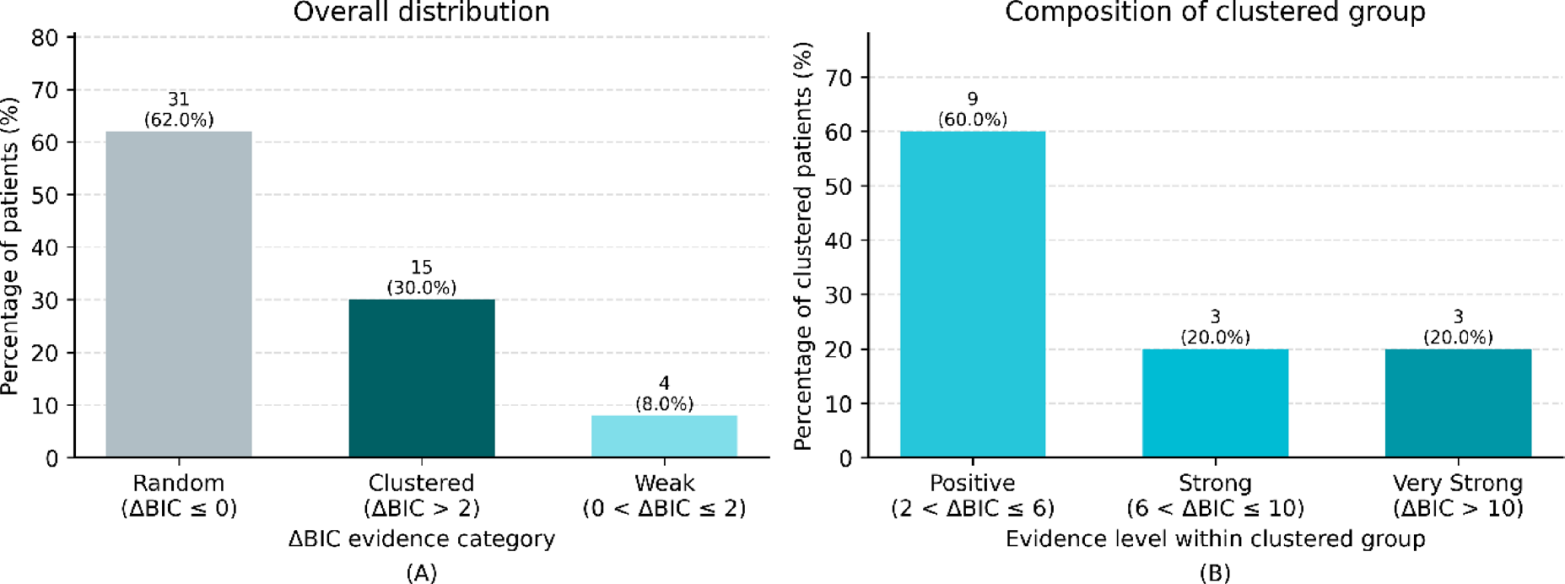
Distribution of patients across ∆BIC evidence categories for seizure clustering. (**A**) shows the proportion of patients classified as Random, Clustered, or Weak, and (**B**) shows the composition of the Clustered group. While the majority of patients have random TOD seizure occurrence, 30% have model-supported evidence of onset clustering.

Figure 3 summarizes the results of the vMM methodology, showing the distribution of patients across the evidence categories defined by Kass and Raftery (1995)^48^:

Figure 3A compares the proportion of patients grouped into the three broad main categories (patients with randomly occurring seizures, with evidence-supported TOD seizure onset clustering, and with weak evidence of clustering). Seeing as the ”clustered” category aggregates different strengths of evidence, we further decomposed it into each evidence level in Fig. 3B. Please see Table S2 of the supplementary materials for the patient-specific vMM results, including the number of clusters, model parameters ($\:\mu\:,\kappa\:,$ and $\:\omega\:$), $\:{\Delta\:}BIC$, and classification of evidence categories.

Figure 3 shows that, among patients with at least 9 lead seizures, model-based statistical evidence for seizure onset TOD patterns was found in 30% of the cohort (15 patients). Of these patients, 3 (6%) exhibited “strong” evidence, and 3 (6%) exhibited “very strong” evidence of seizure onset clustering. An additional subset of 4 (8%) patients displayed “weak” evidence of onset clustering, which, although insufficient to confirm a consistent pattern, does not conclusively support uniform (random) seizure occurrence. The number of patients with statistically supported random seizure occurrence is 62% of the total cohort (31 patients).

**Fig. 4:**
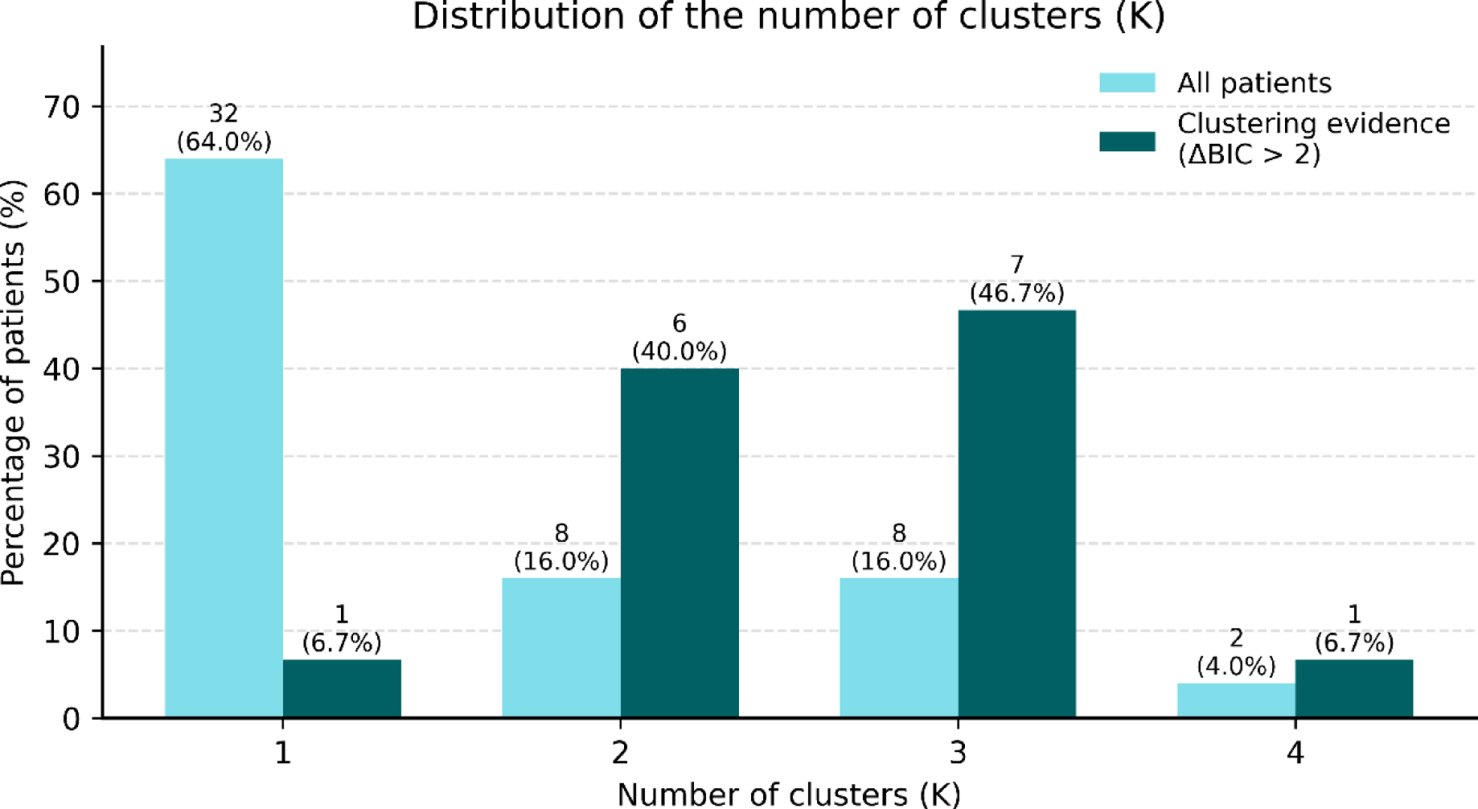
Grouped distribution of the number of clusters (*\:K*) across all patients (light blue) and those with evidence of seizure onset clustering (dark green). Most patients were fit to 1 cluster, but most patients with clustering evidence were found to have 2 or more.

Figure 4 displays the distribution of the number of clusters *\:K* (components of the vMM) and provides a comparison between the values for the entire cohort of patients and the ones with model-based evidence of seizure onset clustering:

Figure 4 shows that one-component models were common in the full primary cohort, but these were mainly associated with patients classified as random. In contrast, patients with evidence of clustering were more frequently described by two- or three-component models. Most “clustered” patients display multiple daily clusters of seizure occurrence, with 40% manifesting two clusters and approximately 47% showing three. This indicates that, when clustering evidence is present, the fitted TOD patterns are often multimodal rather than restricted to a single preferred TOD.

**Fig. 5:**
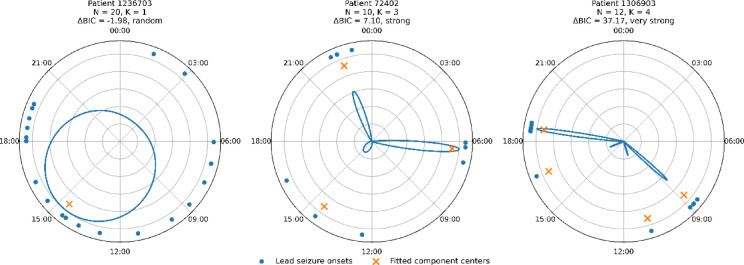
Representative patient-specific von Mises Mixture Model fits. Lead seizure onset times are shown as blue points on the 24-hour circle. The fitted von Mises mixture model density is shown as a blue curve, and the fitted component centers are indicated by orange crosses.

To make the model output more interpretable, Fig. 5 shows representative examples of patient-specific vMM fits across different evidence categories and numbers of selected components. Each panel displays the lead seizure onset times on the 24-hour circle, the fitted vMM density, and the estimated component centers. The radial distance of the fitted curve represents the model-estimated density at each TOD, meaning that larger peaks correspond to times of day where seizure onsets are more likely according to the fitted model. These examples illustrate how the same pipeline can lead to different classifications depending on both the distribution of seizure onsets and the evidence against the uniform model.

Patient 1,236,703 illustrates a case in which the model selected a single component but did not provide evidence against uniform seizure timing ($\:{\Delta\:}BIC=-1.98$). The fitted component is broad, reflecting the fact that seizure onsets are spread over much of the day rather than concentrated around a specific TOD. Therefore, although the model has a preferred direction, this structure is not strong enough to outperform the uniform reference model.

Patient 72,402 shows a three-component solution with strong evidence of TOD clustering ($\:{\Delta\:}BIC=7.10$). In this example, seizure onsets are grouped around multiple TODs, and the fitted density presents three corresponding peaks. The peaks do not all have the same shape, because the components differ in both weight and concentration. This illustrates why the vMM approach is useful for describing multimodal seizure timing patterns rather than forcing the data into a single preferred phase.

Patient 1,306,903 shows a four-component solution with very strong evidence of clustering ($\:{\Delta\:}BIC=37.17$). In this case, most seizure onsets are concentrated in two clear TOD groups, producing two dominant peaks in the fitted density. The model also identifies two additional low-weight components associated with isolated seizure onsets. These isolated components should not be interpreted as strong independent clusters on their own, since single-event components can naturally lead to very narrow fitted concentrations. However, despite the BIC penalty for the larger number of parameters in a four-component model, the overall fit still provides very strong evidence against the uniform model. This suggests that the classification is mainly driven by the highly structured seizure timing distribution as a whole, particularly the dominant TOD groups, rather than by the isolated components alone.

We also wanted to assess whether the strength of evidence for TOD seizure onset patterns differed across anatomical subgroups. Patients were divided by seizure onset localization (temporal or frontal lobe), lateralization (left, right, and bilateral), data acquisition method (scalp EEG and iEEG), and medical center (HdlPS, HUC, and UKLFR), and the corresponding proportions of patients classified as clustered or random. It is important to note that Table 2 reports only patients classified as “clustered” or “random”. Patients with weak evidence of clustering are not shown in this table; therefore, the clustered and random counts do not necessarily sum to the total number of patients in each subgroup. Furthermore, information regarding the localization and lateralization of seizures is not available for every patient. The subgroups and statistical analysis results are summarized in Table 2.

**Table 2:** Distribution of patients with evidence of seizure onset clustering and of random seizure occurrence across multiple subsets of patients.

Variable	Localization		Lateralization			Hospital			Modality	
Subset	Temporal	Frontal	Left-sided	Right-sided	Bilateral	HdlPS	HUC	UKLFR	Scalp EEG	iEEG
No. of patients	21	8	11	19	10	10	7	33	27	23
Clustered	5 23.8%	2 25.0%	4 36.4%	5 26.3%	1 10.0%	5 50.0%	2 28.6%	8 24.6%	8 29.6%	7 30.4%
Random	14 66.7%	6 75.0%	7 63.6%	12 63.2%	8 80.0%	2 20.0%	5 71.4%	24 72.7%	16 59.3%	15 65.2%
p-value	1.000		1.000			0.391			1.000	

There are no statistically significant differences in seizure onset TOD clustering prevalence observed across seizure localization ($\:\mathrm{p}=1.000$), seizure lateralization ($\:\mathrm{p}=1.000$), hospital ($\:\mathrm{p}=0.391$), or recording modality ($\:\mathrm{p}=1.000$) after the Holm-Bonferroni correction for multiple comparisons.

### Exploratory analysis: patients with < 9 seizures

Given the high FPR observed for patients with fewer than 9 lead seizures, the following analysis is reported for completeness and should be considered exploratory rather than confirmatory.

Patients with fewer than 9 seizures were analyzed separately due to the increased FPR observed in this range (Table 1). In this subgroup, clustering classifications are inherently less stable, and results should therefore be interpreted with caution. Nevertheless, we examined these patients to provide a complete overview of the dataset and to assess whether similar qualitative patterns could be observed. This subset contains the remaining 230 patients with 1213 total lead seizures.

**Fig. 6:**
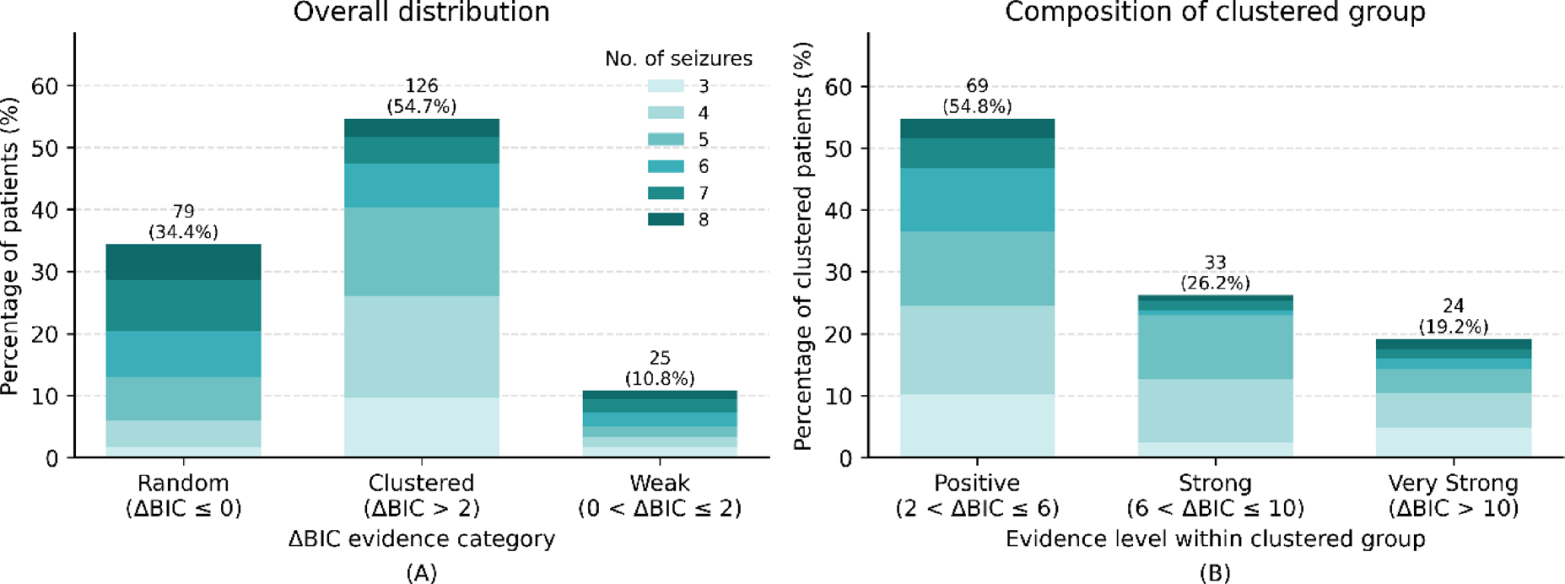
Distribution of patients with fewer than 9 seizures across ∆BIC evidence categories for seizure clustering. (**A**) shows the proportion of patients classified as Random, Clustered, or Weak, and (**B**) shows the composition of the Clustered group. A majority of this group shows model-supported evidence of clustering. However, the results should be treated carefully due to the high False Positive Rates associated with low seizure counts.

Figure 6 summarizes the results of the vMM methodology for the subset of patients with small samples sizes (3 to 8 lead seizures):

Figure 6 has the same structure as Fig. 3, with Fig. 6A showing the distribution across the three main groups, and Fig. 6B displaying the composition of the clustered group. Furthermore, for extra transparency, the bars highlight the contribution of each sample size to the overall distribution of the results among the subset of patients. This Figure shows model-based statistical evidence for non-uniform seizure timing was observed in 55% (126 patients), with 26% (33 patients) exhibiting “strong” and 19% (24 patients) “very strong” evidence of clustering. In contrast, 34% (79 patients) showed model-supported random seizure occurrence throughout the day.

## Discussion

The primary analysis showed that model-supported TOD clustering was present in 15 out of the 50 (30%) patients with a stable FPR, while most patients did not show evidence against uniform seizure timing. This finding supports the presence of patient-specific TOD structure in some cases, but also indicates that such structure is not detectable across the whole cohort. In patients with few recorded seizures, the flexibility of the vMM may lead to an overestimation of non-uniformity, whereas in patients with larger numbers of lead seizures the evidence becomes more conservative. The analysis restricted to patients with smaller samples sizes and higher risk of false positives (less than 9 lead seizures) provides a less stable estimate of seizure onset TOD patterns, as suggested by the FPR (Table 1), but should nonetheless be, at least, reported. Within this subgroup, as shown in Fig. 6, there is a higher proportion of clustered classifications in patients with fewer than 9 lead seizures than in the primary cohort. However, because the FPR analysis showed higher FPRs in this sample-size range, these results should be interpreted as exploratory and cannot be taken as equally stable evidence of patient-specific TOD clustering. Taken together, these findings highlight the strong dependence of model-based classification on the number of observed seizures per patient. The higher prevalence of clustering observed in patients with fewer seizures likely reflects, at least in part, the increased susceptibility of the model to overfitting under sparse data conditions. Nevertheless, the persistence of clustering in the subset of patients with 9 or more seizures indicates that seizure onset TOD patterns are not merely an artifact of statistical modeling, but rather may represent genuine, patient-specific temporal organization that becomes reliably detectable only with adequate data. These results therefore emphasize both the potential relevance of TOD patterns and the methodological constraints inherent to their detection in short presurgical recordings.

Next, when analyzing Fig. 4, considering the subset of patients with statistical evidence for seizure onset TOD patterns (“clustered” subset), we observe a strong tendency toward multimodal distributions, with only 1 patient exhibiting a single cluster of seizure onsets. The relatively frequent occurrence of seizures during presurgical monitoring may also make multiple TOD groups observable within a short admission. However, this should be interpreted in the context of the monitoring setting, where seizure occurrence is clinically facilitated and may not reflect the same temporal structure observed under long-term ambulatory conditions. In contrast, when examining the whole cohort, a different trend emerges: 32 patients were best fit by a one-component model. However, among these 32 patients, 31 showed no model-based evidence of clustering. This convergence of “random” cases toward single-component models is expected, given that the ICL was used to determine the optimal number of components for the vMMs. As shown in Eq. 6, the ICL is built upon the BIC, and, as previously discussed, the BIC (Eq. 5) contains two terms: one that rewards goodness-of-fit, and one that penalizes complexity. When seizure occurrence is effectively random, no model achieves a level of fit sufficient to outweigh the penalty term, leading the pipeline to select the simplest model (one component). This mechanism also explains why the most complex model (four components) is the least frequent. Accordingly, models with higher complexity correspond predominantly to patients with evidence of TOD patterns.

Finally, we further examined whether the prevalence of TOD clustering varied across several clinical and methodological subgroups in Table 2. No statistically significant differences in clustering prevalence were observed across seizure onset localization, seizure lateralization, hospital, or recording modality, suggesting that the likelihood of identifying clustered seizure timing was broadly similar across these patient and recording characteristics. However, patients at HdlPS did show the largest proportion of clustered instances (50% of 10 patients), while those at HUC showed the smallest (29% of 7 patients), despite these differences not being statistically significant, likely due to the small number of samples. This apparent heterogeneity may reflect differences in patient populations, presurgical evaluation strategies, monitoring duration, or local clinical practices, although the present analysis does not allow these factors to be discerned. However, these results should be interpreted cautiously due to small subgroup sizes.

An important dimension of our findings is the possible influence of circadian rhythms on seizure onset clustering. While our focus was on identifying these seizure onset patterns, the presence of distinct TOD clusters in some patients may be compatible with circadian modulation of seizure susceptibility, although this cannot be concluded from TOD information alone. Several studies^15–17,19^ report the existence of circadian cycles in patients with epilepsy. However, the amount of data available for these studies is much greater (ranging from 0.6 to over 9 years) and is recorded under regular conditions, capturing normal daily-life brain dynamics. On the other hand, the data in this study comes from presurgical monitoring, a situation that forces medication withdrawal and higher levels of stress and anxiety in patients. Therefore, the TOD clusters detected in this study should not be interpreted as direct evidence of preserved circadian rhythms or physiological cycles. Instead, they show that seizure onset TOD patterns can persist in some patients even under presurgical monitoring conditions. Such non-uniformity may reflect circadian influences, but it may also be shaped by sleep–wake state, medication changes, hospital routines, seizure provocation strategies, or interactions between these factors. Altogether, these results show that seizure occurrence in a subset of patients exhibits model-supported TOD structure even within presurgical conditions. Determining whether these inpatient TOD patterns correspond to stable circadian rhythms will require datasets with detailed sleep–wake information, medication tapering schedules, and ideally repeated inpatient or outpatient validation.

### Limitations

Several limitations of this study should be acknowledged when interpreting the results. First, although the overall dataset was relatively large, many patients had a small number of lead seizures (a mean of 10 and a median of 7). Mixture modeling is sensitive to sample size, and with such limited data, the ability to detect or exclude TOD patterns is inherently reduced, as was evidenced by the high FPR for sample sizes smaller than 9.

Furthermore, sleep-related factors were not incorporated into the modeling. Sleep and circadian influences on seizure timing are well documented, meaning that some of the detected patterns may reflect sleep-driven modulation rather than purely TOD influence.

Finally, the data reflects the atypical context of presurgical monitoring, during which ASM are frequently altered or withdrawn. Medication changes can significantly alter seizure dynamics, and fluctuations in drug concentrations, particularly for ASM with short half-lives, may introduce changes that either mimic or obscure underlying physiological rhythms^52^. Seeing as medication regimens, plasma levels, and dosing cycles were not incorporated into the analysis, we cannot determine the extent to which the observed TOD patterns were influenced by ASM changes rather than underlying cyclical processes.

## Conclusions

This study used a circular mixture-model framework to retrospectively characterize patient-specific TOD seizure onset patterns during presurgical monitoring. In patients with at least 9 lead seizures, model-supported evidence of TOD clustering was found in 30%, showing that non-uniform seizure timing can be detected even in the clinically altered context of presurgical monitoring. Considering the remaining patients, over half presented clustering prevalence. However, the false positive analysis showed that the method is highly sensitive to the number of lead seizures available per patient, making classifications in patients with fewer than 9 seizures simply exploratory and less stable. Therefore, these findings should be interpreted as retrospective evidence of non-uniform TOD seizure timing during inpatient monitoring, rather than as evidence of stable circadian seizure cycles or clinically actionable seizure forecasts.

Future work should integrate the EEG data with detailed information on medication changes and sleep–wake states to more fully characterize the mechanisms underlying these TOD patterns. In addition, a valuable future direction would be to compare patients’ everyday cycles to ones found in these settings. If a consistent relationship between inpatient and outpatient patterns can be established, short-term recordings in presurgical monitoring-like conditions could become a practical way to characterize individual seizure cycles and, ultimately, help modulate seizure risk based on limited monitoring.

## Supplementary Information

Below is the link to the electronic supplementary material.

Supplementary Material 1

## Data Availability

This study uses data from the EPILEPSIAE database. The data was provided under license for this study by the EPILEPSIAE Consortium. Therefore, it is not publicly available. All the code used in this study is available at https://github.com/GoncaloSantosCosta/Circular-mixture-model-based-characterization-of-TOD-seizure-patterns-during-presurgical-monitoring.
