# Supplementary material for: Circular mixture model-based characterization of time-of-day seizure patterns during presurgical monitoring
Source: Sci Rep. 2026 May 19;16:24234. doi: 10.1038/s41598-026-53249-1 (PMC13444133; doi:10.1038/s41598-026-53249-1)
Supplement: Supplementary file 1 [file 41598_2026_53249_MOESM1_ESM.pdf]

Supplementary material for:

Circular mixture model-based characterization of time-of-day seizure patterns during presurgical monitoring

Gonçalo Costa, Matthias Dümpelmann, César Teixeira

1 Patient metadata

Table S1 contains information on each patient information (gender, age, admission hospital, number of lead seizures, and seizure onset and lateralization). The data was obtained from the EPILEPSIAE database[1].

Table S1: Information for the 285 studied patients.

Patient ID	Sex	Age	Hospital	Number of lead seizures	Seizure onset location	Seizure onset lateralization	Modality
102	m	36	UKLFR	11	-	bilateral	scalp EEG
200	f	53	HUC	6	temporal	right	scalp EEG
202	m	24	UKLFR	5	-	left	scalp EEG
300	m	20	HUC	7	frontal	-	scalp EEG
400	f	36	HUC	6	temporal	left	scalp EEG
402	f	55	UKLFR	5	temporal	bilateral	scalp EEG
500	m	57	HUC	7	temporal	right	scalp EEG
600	f	50	HUC	8	temporal	-	scalp EEG
700	m	21	HUC	12	temporal	left	iEEG
800	f	38	HUC	7	temporal	left	scalp EEG
1200	f	40	HUC	10	temporal	right	scalp EEG
1300	f	36	HUC	5	temporal	left	scalp EEG
1400	m	42	HUC	5	temporal	left	scalp EEG
1500	m	50	HUC	6	temporal	right	scalp EEG
1600	m	33	HUC	6	temporal	left	scalp EEG
1700	m	40	HUC	8	-	-	scalp EEG
2000	f	37	HUC	4	temporal	right	scalp EEG
2100	m	20	HUC	8	temporal	left	scalp EEG
2200	m	21	HUC	9	frontal	right	scalp EEG
2300	m	49	HUC	7	temporal	left	scalp EEG
2600	f	41	HUC	5	temporal	right	scalp EEG
2700	f	20	HUC	9	occipital	left	scalp EEG
2800	f	18	HUC	4	frontal	-	scalp EEG
2900	f	21	HUC	8	frontal	-	scalp EEG
3300	m	29	HUC	6	-	-	scalp EEG
3500	m	34	HUC	4	temporal	right	scalp EEG
3600	m	10	HUC	13	-	-	scalp EEG
3700	f	35	HUC	5	parietal	left	scalp EEG
3800	m	57	HUC	4	parietal	left	iEEG
4000	m	55	HUC	5	temporal	left	scalp EEG
4200	f	34	HUC	3	temporal	-	scalp EEG
4400	m	25	HUC	4	temporal	right	scalp EEG
4500	m	49	HUC	4	temporal	left	scalp EEG
4600	m	16	HUC	4	temporal	right	scalp EEG
4700	m	57	HUC	4	temporal	right	scalp EEG
4900	f	32	HUC	4	temporal	left	scalp EEG
5100	m	15	HUC	6	temporal	right	scalp EEG
5200	m	57	HUC	5	temporal	-	scalp EEG
5300	m	55	HUC	4	frontal	right	iEEG
5500	f	35	HUC	3	temporal	-	scalp EEG

Table S1 – *Continued from previous page*

Patient ID	Sex	Age	Hospital	Number of lead seizures	Seizure onset location	Seizure onset lateralization	Modality
5800	f	26	HUC	6	temporal	-	scalp EEG
5900	f	27	HUC	5	temporal	right	scalp EEG
6000	m	16	HUC	5	temporal	left	scalp EEG
6100	f	27	HUC	11	temporal	-	scalp EEG
6200	f	37	HUC	8	temporal	left	scalp EEG
6300	m	33	HUC	5	temporal	-	scalp EEG
6400	f	31	HUC	2	-	-	scalp EEG
6500	m	27	HUC	11	-	-	scalp EEG
6600	f	44	HUC	5	temporal	right	scalp EEG
6700	m	35	HUC	6	temporal	right	scalp EEG
6800	m	30	HUC	4	temporal	left	scalp EEG
6900	m	38	HUC	5	-	-	scalp EEG
7000	f	36	HUC	4	temporal	-	scalp EEG
7200	m	41	HUC	4	temporal	-	scalp EEG
7300	m	18	HUC	7	-	-	scalp EEG
7302	f	52	UKLFR	7	-	bilateral	scalp EEG
7500	f	29	HUC	3	temporal	left	scalp EEG
7700	m	37	HUC	7	-	-	scalp EEG
7800	m	55	HUC	4	temporal	-	scalp EEG
8100	m	16	HUC	5	temporal	-	scalp EEG
8902	f	67	UKLFR	5	temporal	left	scalp EEG
11002	m	41	UKLFR	6	temporal	right	scalp EEG
11502	m	34	UKLFR	17	temporal	right	iEEG
12702	f	16	UKLFR	6	-	-	iEEG
13902	f	53	UKLFR	6	temporal	bilateral	iEEG
16202	f	46	UKLFR	7	temporal	bilateral	scalp EEG
19202	m	33	UKLFR	8	-	bilateral	scalp EEG
21602	m	44	UKLFR	9	-	bilateral	scalp EEG
21902	m	47	UKLFR	4	temporal	left	scalp EEG
22602	m	26	UKLFR	6	frontal	left	scalp EEG
23902	m	36	UKLFR	5	temporal	left	scalp EEG
25302	f	37	UKLFR	5	temporal	bilateral	iEEG
26102	m	65	UKLFR	5	temporal	left	scalp EEG
26402	f	48	UKLFR	5	temporal	-	iEEG
27302	f	3	UKLFR	6	temporal	bilateral	iEEG
30002	m	48	UKLFR	10	-	bilateral	scalp EEG
30802	m	28	UKLFR	8	temporal	bilateral	scalp EEG
32202	m	64	UKLFR	8	-	-	scalp EEG
32502	m	46	UKLFR	6	-	-	scalp EEG
32702	f	62	UKLFR	5	temporal	bilateral	scalp EEG
37502	m	35	UKLFR	14	-	bilateral	iEEG
38402	f	50	UKLFR	13	frontal	right	iEEG
44202	m	21	UKLFR	14	temporal	right	iEEG
45402	f	41	UKLFR	4	temporal	bilateral	scalp EEG
46702	f	15	UKLFR	5	temporal	right	scalp EEG
50802	m	43	UKLFR	5	temporal	left	scalp EEG
51002	f	46	UKLFR	8	-	bilateral	scalp EEG
52302	f	61	UKLFR	5	temporal	left	scalp EEG
52303	m	36	HdlPS	5	-	-	iEEG
53402	m	39	UKLFR	5	temporal	bilateral	scalp EEG
54802	m	17	UKLFR	16	temporal	left	iEEG
55202	f	17	UKLFR	9	temporal	-	scalp EEG
56402	m	47	UKLFR	6	temporal	bilateral	scalp EEG
56502	m	13	UKLFR	6	temporal	left	iEEG
58302	f	22	UKLFR	15	temporal	left	iEEG
58602	m	32	UKLFR	12	temporal	left	scalp EEG
59002	m	18	UKLFR	9	temporal	bilateral	iEEG
59102	m	47	UKLFR	5	temporal	right	scalp EEG

Table S1 – *Continued from previous page*

Patient ID	Sex	Age	Hospital	Number of lead seizures	Seizure onset location	Seizure onset lateralization	Modality
60002	m	55	UKLFR	7	temporal	bilateral	scalp EEG
62002	m	42	UKLFR	5	temporal	right	iEEG
63502	f	63	UKLFR	8	temporal	bilateral	iEEG
64702	m	51	UKLFR	5	temporal	right	scalp EEG
70102	f	46	UKLFR	6	temporal	bilateral	iEEG
70202	f	29	UKLFR	6	temporal	left	scalp EEG
70302	m	18	UKLFR	11	temporal	right	scalp EEG
70402	f	37	UKLFR	5	temporal	bilateral	scalp EEG
70502	m	18	UKLFR	8	temporal	left	scalp EEG
70602	m	35	UKLFR	7	-	-	iEEG
70702	m	26	UKLFR	6	frontal	right	scalp EEG
70802	f	45	UKLFR	11	-	-	scalp EEG
70902	f	28	UKLFR	6	-	-	iEEG
71002	f	30	UKLFR	6	temporal	right	scalp EEG
71102	f	31	UKLFR	6	temporal	bilateral	scalp EEG
71202	m	41	UKLFR	7	temporal	left	scalp EEG
71302	m	37	UKLFR	4	central	left	scalp EEG
71402	f	42	UKLFR	3	-	-	scalp EEG
71502	f	18	UKLFR	5	-	bilateral	scalp EEG
71602	m	45	UKLFR	11	-	bilateral	iEEG
71702	m	29	UKLFR	7	-	-	scalp EEG
71802	f	46	UKLFR	5	-	-	iEEG
71902	f	21	UKLFR	4	-	-	scalp EEG
72002	f	33	UKLFR	3	temporal	bilateral	iEEG
72102	m	25	UKLFR	4	temporal	bilateral	iEEG
72202	f	27	UKLFR	11	temporal	bilateral	iEEG
72302	m	21	UKLFR	6	-	left	scalp EEG
72402	f	36	UKLFR	10	-	-	scalp EEG
72502	f	60	UKLFR	7	temporal	bilateral	iEEG
72602	m	55	UKLFR	8	temporal	right	scalp EEG
72702	f	56	UKLFR	8	-	-	scalp EEG
72802	f	36	UKLFR	5	-	-	scalp EEG
72902	f	15	UKLFR	4	-	-	scalp EEG
73002	m	32	UKLFR	5	temporal	bilateral	iEEG
73102	f	40	UKLFR	2	temporal	right	iEEG
75102	f	2	UKLFR	6	-	right	scalp EEG
75202	m	13	UKLFR	7	temporal	right	scalp EEG
79502	f	35	UKLFR	8	-	left	scalp EEG
80602	f	34	UKLFR	5	frontal	left	scalp EEG
80702	f	22	UKLFR	7	temporal	-	scalp EEG
81102	m	41	UKLFR	7	temporal	right	scalp EEG
81402	f	65	UKLFR	7	frontal	left	scalp EEG
81802	f	27	UKLFR	8	temporal	left	iEEG
85202	f	54	UKLFR	7	temporal	left	scalp EEG
86202	f	16	UKLFR	6	frontal	left	iEEG
91602	m	23	UKLFR	26	temporal	left	iEEG
92102	m	38	UKLFR	5	parietal	right	scalp EEG
92202	m	39	UKLFR	15	-	left	iEEG
93402	m	67	UKLFR	5	temporal	left	scalp EEG
93902	m	50	UKLFR	7	temporal	right	scalp EEG
94402	f	37	UKLFR	8	temporal	right	scalp EEG
95202	f	50	UKLFR	7	temporal	left	scalp EEG
95802	f	14	UKLFR	8	temporal	left	iEEG
96002	m	58	UKLFR	7	temporal	bilateral	scalp EEG
97002	m	15	UKLFR	11	temporal	right	iEEG
98102	m	36	UKLFR	5	temporal	left	scalp EEG
98202	m	39	UKLFR	9	temporal	right	scalp EEG
100002	m	35	UKLFR	12	-	-	scalp EEG

Table S1 – *Continued from previous page*

Patient ID	Sex	Age	Hospital	Number of lead seizures	Seizure onset location	Seizure onset lateralization	Modality
100302	f	42	UKLFR	2	-	bilateral	scalp EEG
101702	m	52	UKLFR	5	temporal	bilateral	scalp EEG
102202	m	17	UKLFR	12	temporal	left	scalp EEG
103002	f	31	UKLFR	6	occipital	right	scalp EEG
103802	f	17	UKLFR	7	-	-	scalp EEG
104602	f	17	UKLFR	5	temporal	left	scalp EEG
107302	f	47	UKLFR	12	temporal	right	iEEG
107702	f	29	UKLFR	8	temporal	right	iEEG
107802	m	11	UKLFR	11	frontal	right	iEEG
108402	f	48	UKLFR	19	frontal	left	iEEG
109202	f	49	UKLFR	5	frontal	bilateral	scalp EEG
109502	m	50	UKLFR	6	temporal	bilateral	scalp EEG
109602	f	32	UKLFR	6	temporal	left	iEEG
110602	m	56	UKLFR	6	temporal	right	scalp EEG
111902	m	42	UKLFR	6	central	left	scalp EEG
112402	f	32	UKLFR	13	frontal	right	scalp EEG
112502	f	11	UKLFR	10	temporal	right	iEEG
112802	m	52	UKLFR	6	temporal	left	scalp EEG
113902	f	29	UKLFR	12	temporal	right	scalp EEG
114602	m	22	UKLFR	7	-	bilateral	iEEG
114702	f	22	UKLFR	15	temporal	right	scalp EEG
114902	f	16	UKLFR	6	temporal	bilateral	scalp EEG
115002	f	32	UKLFR	6	-	bilateral	iEEG
115102	m	31	UKLFR	11	frontal	right	scalp EEG
115202	f	32	UKLFR	9	-	bilateral	scalp EEG
123902	f	25	UKLFR	6	temporal	bilateral	scalp EEG
125002	m	39	UKLFR	7	-	-	scalp EEG
132502	f	18	UKLFR	11	-	left	iEEG
1233703	f	42	HdlPS	3	-	right	scalp EEG
1233803	f	29	HdlPS	3	temporal	left	scalp EEG
1234303	m	30	HdlPS	5	temporal	left	scalp EEG
1235003	f	42	HdlPS	4	temporal	left	scalp EEG
1235103	m	19	HdlPS	6	temporal	-	scalp EEG
1235903	f	21	HdlPS	7	temporal	right	iEEG
1236703	m	48	HdlPS	20	frontal	left	iEEG
1241003	m	38	HdlPS	5	frontal	left	iEEG
1242003	m	27	HdlPS	7	temporal	left	iEEG
1242703	f	39	HdlPS	5	temporal	right	iEEG
1243603	m	34	HdlPS	5	temporal	right	iEEG
1244803	f	31	HdlPS	6	temporal	left	iEEG
1245203	m	38	HdlPS	8	temporal	right	iEEG
1256303	m	38	HdlPS	3	occipital	-	iEEG
1259203	m	30	HdlPS	5	-	left	iEEG
1270403	m	40	HdlPS	6	temporal	left	iEEG
1272703	f	23	HdlPS	5	temporal	right	iEEG
1299403	m	45	HdlPS	3	temporal	left	scalp EEG
1300003	f	22	HdlPS	4	temporal	right	scalp EEG
1300503	f	23	HdlPS	4	-	left	iEEG
1305803	m	19	HdlPS	3	-	left	scalp EEG
1306003	m	29	HdlPS	4	temporal	-	scalp EEG
1306203	m	47	HdlPS	10	-	-	scalp EEG
1306903	m	42	HdlPS	12	-	-	scalp EEG
1307003	m	25	HdlPS	3	temporal	right	scalp EEG
1307103	f	37	HdlPS	8	-	-	scalp EEG
1307403	m	19	HdlPS	6	temporal	-	scalp EEG
1307503	f	24	HdlPS	7	-	-	scalp EEG
1307803	m	19	HdlPS	4	-	-	scalp EEG
1308403	m	18	HdlPS	3	temporal	-	scalp EEG

Table S1 – *Continued from previous page*

Patient ID	Sex	Age	Hospital	Number of lead seizures	Seizure onset location	Seizure onset lateralization	Modality
1308503	m	19	HdlPS	4	-	left	scalp EEG
1308603	m	35	HdlPS	4	temporal	-	scalp EEG
1308903	f	29	HdlPS	8	temporal	right	iEEG
1309803	m	19	HdlPS	5	-	left	scalp EEG
1310403	m	26	HdlPS	4	temporal	right	iEEG
1310803	f	38	HdlPS	4	temporal	left	scalp EEG
1311003	f	57	HdlPS	3	temporal	left	scalp EEG
1311203	f	48	HdlPS	13	temporal	bilateral	iEEG
1312603	f	22	HdlPS	3	-	right	scalp EEG
1312703	f	54	HdlPS	4	temporal	bilateral	scalp EEG
1312803	m	35	HdlPS	3	-	left	scalp EEG
1312903	f	19	HdlPS	4	frontal	left	scalp EEG
1313003	f	44	HdlPS	7	temporal	bilateral	scalp EEG
1313403	f	46	HdlPS	4	temporal	bilateral	scalp EEG
1313903	m	27	HdlPS	4	-	-	scalp EEG
1314103	f	17	HdlPS	4	-	bilateral	scalp EEG
1314703	m	32	HdlPS	3	temporal	left	scalp EEG
1314803	f	23	HdlPS	7	-	left	scalp EEG
1315003	m	43	HdlPS	4	-	right	scalp EEG
1315203	m	21	HdlPS	4	-	right	scalp EEG
1315403	m	19	HdlPS	4	temporal	bilateral	scalp EEG
1316003	m	32	HdlPS	3	-	-	scalp EEG
1316303	f	42	HdlPS	3	temporal	left	scalp EEG
1316403	m	27	HdlPS	7	-	-	scalp EEG
1316503	m	36	HdlPS	4	temporal	right	scalp EEG
1317003	f	32	HdlPS	3	-	left	scalp EEG
1317203	m	37	HdlPS	3	temporal	left	scalp EEG
1317303	f	54	HdlPS	4	temporal	bilateral	scalp EEG
1317403	m	23	HdlPS	3	-	right	scalp EEG
1317903	m	23	HdlPS	4	-	-	scalp EEG
1318803	m	41	HdlPS	3	temporal	bilateral	scalp EEG
1319103	m	26	HdlPS	4	-	bilateral	scalp EEG
1319203	m	28	HdlPS	7	-	right	scalp EEG
1320303	m	48	HdlPS	3	temporal	right	scalp EEG
1320503	m	16	HdlPS	3	temporal	left	scalp EEG
1320603	f	40	HdlPS	3	temporal	right	scalp EEG
1320903	f	29	HdlPS	4	temporal	bilateral	scalp EEG
1321003	m	24	HdlPS	3	temporal	right	scalp EEG
1321103	f	38	HdlPS	2	temporal	left	scalp EEG
1321803	f	27	HdlPS	5	temporal	bilateral	scalp EEG
1321903	m	41	HdlPS	5	-	left	scalp EEG
1322103	m	42	HdlPS	11	-	bilateral	iEEG
1322703	f	45	HdlPS	3	temporal	left	scalp EEG
1322803	m	18	HdlPS	4	-	right	scalp EEG
1322903	f	23	HdlPS	4	temporal	right	iEEG
1323803	m	30	HdlPS	5	temporal	right	scalp EEG
1324103	m	45	HdlPS	4	-	bilateral	scalp EEG
1324503	f	43	HdlPS	11	-	right	iEEG
1324803	f	46	HdlPS	9	central	right	scalp EEG
1324903	f	22	HdlPS	10	central	right	scalp EEG
1325003	f	20	HdlPS	1	-	right	scalp EEG
1325103	f	37	HdlPS	4	temporal	right	scalp EEG
1325403	m	37	HdlPS	4	temporal	left	scalp EEG
1325603	f	37	HdlPS	4	temporal	bilateral	scalp EEG
1325903	m	41	HdlPS	5	-	bilateral	scalp EEG
1326003	f	24	HdlPS	7	frontal	-	scalp EEG
1326103	f	34	HdlPS	4	temporal	right	scalp EEG
1326503	m	28	HdlPS	3	-	bilateral	scalp EEG

Table S1 – *Continued from previous page*

Patient ID	Sex	Age	Hospital	Number of lead seizures	Seizure onset location	Seizure onset lateralization	Modality
1327403	m	50	HdlPS	7	temporal	left	scalp EEG
1327603	f	23	HdlPS	7	-	-	iEEG
1327903	f	21	HdlPS	8	frontal	right	scalp EEG
1328403	m	20	HdlPS	4	temporal	left	scalp EEG
1328603	m	42	HdlPS	8	temporal	left	scalp EEG
1328803	m	46	HdlPS	5	frontal	right	scalp EEG
1328903	m	32	HdlPS	5	temporal	-	scalp EEG
1329003	m	36	HdlPS	9	frontal	right	iEEG
1329303	f	23	HdlPS	3	-	bilateral	scalp EEG
1329503	f	18	HdlPS	4	frontal	-	scalp EEG
1330103	f	18	HdlPS	3	temporal	left	scalp EEG
1330203	f	31	HdlPS	4	temporal	right	scalp EEG
1330903	m	36	HdlPS	9	-	-	scalp EEG

Sex: female (f), male (m); Hospital: Hôpital de la Pitié-Salpêtrière (HdlPS), University Hospital of Coimbra (HUC), Universitätsklinikum Freiburg (UKLFR); - means the information is unknown.

2 Maximum-likelihood estimation

This section describes the maximum-likelihood estimation framework used in this study, implemented in the *vonMisesMixture*[2] Python package.

Considering $\bar{S}$ and $\bar{C}$ as the average sine and cosine values for the set of seizure onset phases θ, respectively, the mean direction $\hat{\mu}$ was calculated using:

$$tan(\hat{\mu}) = \frac{\bar{S}}{\bar{C}}. \tag{1}$$

The concentration $\hat{\kappa}$ was calculated by solving:

$$\frac{I_1(\hat{\kappa})}{I_0(\hat{\kappa})} + \bar{\sigma} = 1, \tag{2}$$

with the circular variance of the distribution $\bar{\sigma} = 1 - \sqrt{\bar{S}^2 + \bar{C}^2}$.

3 Expectation-Maximization algorithm

This section describes the Expectation-Maximization (EM) algorithm used in this study, implemented in the *vonMisesMixture*[2] Python package.

During the Expectation (E)-step, the algorithm computes the posterior responsibilities at each iteration from the parameter values of the previous iteration, after randomly initializing the parameters on the first iteration. The posterior probability (responsibility) that each seizure onset phase θ_i belongs to each component k is estimated by:

$$\tau_{ik} = \frac{\omega_k f_{vM}(\theta_i|\mu_k, \kappa_k)}{\sum_{j=1}^{K} \omega_j f_{vM}(\theta_i|\mu_j, \kappa_j)}. \tag{3}$$

Then, in the Maximization (M)-step, new values for the parameters μ_k, κ_k, and ω_k are estimated. The weights ω_k were updated via the mean of the responsibilities:

$$\hat{\omega}_k = \frac{1}{m} \sum_i \tau_{ik}. \tag{4}$$

The mean directions μ_k were updated by:

$$tan(\hat{\mu}_k) = \frac{\sum_i \tau_{ik}\, sin(\theta_i)}{\sum_i \tau_{ik}\, cos(\theta_i)}. \tag{5}$$

The concentrations κ_k were updated by solving:

$$\frac{I_1(\hat{\kappa}_k)}{I_0(\hat{\kappa}_k)} = \frac{\sum_i \tau_{ik} \cos(\theta_i - \hat{\mu}_k)}{\sum_i \tau_{ik}}. \tag{6}$$

4 Results obtained for all patients

Table S2 contains the number of identified clusters (K), model parameters (mean direction μ, concentration κ, and mixture weights ω), ΔBIC, and classification of evidence categories.

Table S2: Full results for all 280 patients.

Patient ID	N	K	μ (in hours and minutes)	κ	ω	ΔBIC	Evidence categories
102	11	1	08:38	0.33	1.00	-4,21	random
200	6	1	03:24	0.23	1.00	-3,42	random
202	5	2	06:29; 00:37	690.95; 1.70	0.39; 0.61	4,86	positive
300	7	4	16:13; 11:33; 06:26; 21:49	209.16; 157.12; 694.49; 8.23	0.29; 0.29; 0.14; 0.29	-2,14	random
400	6	1	15:15	0.82	1.00	-1,78	random
402	5	2	08:55; 23:26	704.29; 4.07	0.40; 0.60	8,14	strong
500	7	2	10:20; 20:25	7.48; 36.44	0.43; 0.57	6,71	strong
600	8	1	12:13	0.57	1.00	-2,92	random
700	12	1	10:14	0.45	1.00	-3,79	random
800	7	3	23:05; 14:26; 04:42	661.58; 5.07; 61.14	0.14; 0.43; 0.43	0,55	weak
1200	10	1	09:59	0.25	1.00	-4,3	random
1300	5	1	00:30	1.12	1.00	-0,68	random
1400	5	1	18:48	0.54	1.00	-2,52	random
1500	6	1	11:51	0.65	1.00	-2,42	random
1600	6	1	08:48	0.62	1.00	-2,52	random
1700	8	2	05:42; 17:02	409.74; 0.83	0.37; 0.63	1,37	weak
2000	4	2	09:32; 15:08	31.48; 68.83	0.50; 0.50	6,19	strong
2100	8	3	08:27; 00:17; 13:15	168.08; 10.19; 26.02	0.37; 0.25; 0.38	2,37	positive
2200	9	1	02:46	0.68	1.00	-2,48	random
2300	7	4	07:42; 03:37; 11:30; 20:02	704.88; 331.42; 704.89; 12.14	0.14; 0.29; 0.14; 0.43	0,27	weak
2600	5	1	23:32	0.20	1.00	-3,12	random
2700	9	3	06:17; 16:05; 01:53	108.44; 25.51; 139.42	0.44; 0.33; 0.22	9,12	strong
2800	4	3	16:27; 13:01; 09:27	672.40; 672.40; 671.11	0.25; 0.25; 0.50	12,92	very strong
2900	8	2	17:33; 08:55	2.47; 28.59	0.63; 0.37	-0,95	random
3300	6	1	09:29	0.32	1.00	-3,28	random
3500	4	1	06:23	0.10	1.00	-2,75	random
3600	13	2	05:19; 18:32	132.93; 1.78	0.30; 0.70	3,38	positive
3700	5	3	16:26; 00:07; 10:38	659.01; 68.67; 659.02	0.20; 0.60; 0.20	9,46	strong
3800	4	1	10:12	0.58	1.00	-2,14	random
4000	5	1	21:45	0.27	1.00	-3,03	random
4200	3	1	12:29	1.43	1.00	0,02	weak
4400	4	3	09:11; 18:49; 14:32	208.45; 682.58; 682.58	0.50; 0.25; 0.25	9,66	strong
4500	4	3	20:42; 10:08; 14:22	5.39; 699.56; 699.55	0.50; 0.25; 0.25	2,2	positive
4600	4	3	15:48; 10:45; 05:07	670.56; 73.25; 670.55	0.25; 0.50; 0.25	7,52	strong
4700	4	2	04:58; 14:25	5.16; 510.17	0.50; 0.50	6,39	strong
4900	4	3	17:53; 06:44; 23:42	79.89; 696.38; 696.38	0.50; 0.25; 0.25	7,69	strong
5100	6	1	09:50	0.34	1.00	-3,24	random
5200	5	3	06:02; 17:32; 02:36	705.22; 2.53; 705.22	0.20; 0.60; 0.20	-1,17	random
5300	4	3	18:27; 03:26; 11:38	667.45; 3.76; 667.45	0.25; 0.50; 0.25	1,24	weak
5500	3	2	02:25; 18:43	2.85; 706.46	0.67; 0.33	2,35	positive
5800	6	2	19:40; 13:13	204.24; 2.96	0.33; 0.67	2,61	positive
5900	5	1	18:27	0.97	1.00	-1,23	random
6000	5	1	02:06	0.82	1.00	-1,74	random
6100	11	1	19:05	0.13	1.00	-4,71	random
6200	8	3	21:43; 09:14; 13:42	12.64; 16.32; 699.03	0.38; 0.38; 0.25	1,95	weak
6300	5	2	10:13; 20:09	3.32; 695.89	0.80; 0.20	2,62	positive
6500	11	1	15:09	0.67	1.00	-2,54	random
6600	5	3	15:27; 05:05; 10:42	82.70; 20.11; 702.16	0.40; 0.40; 0.20	3,07	positive
6700	6	2	06:18; 14:57	20.88; 10.69	0.50; 0.50	3,75	positive
6800	4	2	08:24; 17:18	1.97; 402.73	0.50; 0.50	3,5	positive
6900	5	1	16:52	1.65	1.00	1,29	weak
7000	4	3	22:45; 06:21; 12:15	4.12; 695.85; 695.85	0.50; 0.25; 0.25	1,55	weak
7200	4	1	05:51	0.88	1.00	-1,41	random
7300	7	1	03:54	0.25	1.00	-3,67	random
7302	7	1	00:49	1.03	1.00	-0,81	random
7500	3	2	22:02; 15:07	660.72; 659.57	0.33; 0.67	14,73	very strong
7700	7	1	02:22	0.40	1.00	-3,36	random
7800	4	1	13:47	0.17	1.00	-2,71	random

Table S2 – *Continued from previous page*

Patient ID	N	K	μ (in hours and minutes)	κ	ω	ΔBIC	Evidence categories
8100	5	2	18:50; 11:08	23.21; 50.68	0.60; 0.40	6,61	strong
8902	5	2	05:24; 23:50	296.16; 37.16	0.40; 0.60	11,59	very strong
11002	6	4	15:29; 20:06; 07:28; 00:00	441.59; 666.07; 21.44; 666.07	0.33; 0.17; 0.33; 0.17	2,2	positive
11502	17	1	06:03	0.15	1.00	-5,48	random
12702	6	2	17:17; 05:03	8.02; 4.03	0.50; 0.50	-2,5	random
13902	6	1	23:07	0.81	1.00	-1,83	random
16202	7	3	19:08; 04:50; 13:51	142.63; 18.07; 701.45	0.29; 0.57; 0.14	5,77	positive
19202	8	1	02:01	1.40	1.00	1,56	weak
21602	9	2	02:35; 13:39	112.17; 1.50	0.33; 0.67	-0,68	random
21902	4	2	18:25; 07:46	3.61; 17.51	0.50; 0.50	-1,26	random
22602	6	1	04:36	1.28	1.00	0,19	weak
23902	5	3	10:48; 21:34; 16:48	58.79; 28.33; 656.80	0.40; 0.40; 0.20	3,03	positive
25302	5	3	10:16; 16:56; 06:03	54.94; 609.87; 606.76	0.40; 0.20; 0.40	10,95	very strong
26102	5	3	08:13; 15:32; 12:04	127.39; 667.10; 71.86	0.40; 0.20; 0.40	6,44	strong
26402	5	3	23:41; 19:13; 10:28	474.37; 705.01; 36.84	0.40; 0.20; 0.40	8,58	strong
27302	6	2	04:17; 17:45	697.00; 1.85	0.33; 0.67	3,82	positive
30002	10	1	03:31	0.84	1.00	-1,5	random
30802	8	1	04:50	1.91	1.00	4,49	positive
32202	8	1	07:08	1.06	1.00	-0,46	random
32502	6	2	09:20; 20:33	3.40; 63.24	0.33; 0.67	7,03	strong
32702	5	2	09:38; 17:03	24.89; 620.66	0.80; 0.20	11,34	very strong
37502	14	1	07:48	0.11	1.00	-5,19	random
38402	13	3	00:16; 06:52; 14:00	24.14; 22.77; 682.75	0.46; 0.46; 0.08	11,8	very strong
44202	14	1	17:01	0.47	1.00	-3,81	random
45402	4	3	15:05; 08:11; 01:49	580.92; 582.86; 582.86	0.50; 0.25; 0.25	11,82	very strong
46702	5	3	16:32; 06:31; 02:02	42.95; 231.32; 702.49	0.40; 0.40; 0.20	6,81	strong
50802	5	1	03:01	0.94	1.00	-1,31	random
51002	8	1	09:49	0.63	1.00	-2,67	random
52302	5	1	09:19	0.91	1.00	-1,43	random
52303	5	2	17:51; 03:09	2.77; 664.99	0.60; 0.40	6,33	strong
53402	5	2	07:48; 19:06	7.29; 663.59	0.60; 0.40	9,72	strong
54802	16	1	15:05	0.48	1.00	-3,77	random
55202	9	3	07:36; 09:56; 16:43	43.41; 608.64; 6.51	0.22; 0.22; 0.56	3,46	positive
56402	6	1	07:05	0.97	1.00	-1,19	random
56502	6	1	11:41	0.20	1.00	-3,46	random
58302	15	2	09:43; 18:40	44.21; 2.43	0.33; 0.67	5,06	positive
58602	12	1	04:14	0.13	1.00	-4,87	random
59002	9	1	00:45	0.68	1.00	-2,5	random
59102	5	3	09:26; 15:42; 20:32	55.66; 682.22; 32.56	0.40; 0.20; 0.40	3,24	positive
60002	7	1	03:26	1.15	1.00	-0,18	random
62002	5	2	21:58; 07:29	5.87; 665.41	0.80; 0.20	5,32	positive
63502	8	1	02:49	1.05	1.00	-0,5	random
64702	5	2	04:02; 17:45	120.01; 2.60	0.40; 0.60	1,01	weak
70102	6	3	21:41; 08:37; 13:24	693.31; 93.80; 22.10	0.17; 0.50; 0.33	5,85	positive
70202	6	1	22:00	0.43	1.00	-3,06	random
70302	11	3	17:00; 23:44; 07:22	3.50; 41.44; 25.08	0.18; 0.54; 0.27	2,31	positive
70402	5	2	13:16; 06:25	2.10; 663.20	0.60; 0.40	4,83	positive
70502	8	1	10:42	0.22	1.00	-3,96	random
70602	7	1	14:05	1.87	1.00	3,48	positive
70702	6	1	00:43	0.76	1.00	-2,04	random
70802	11	1	11:45	0.52	1.00	-3,38	random
70902	6	3	19:16; 07:20; 13:03	4.27; 633.21; 673.46	0.50; 0.33; 0.17	2,42	positive
71002	6	3	07:04; 12:31; 16:49	13.16; 681.67; 681.66	0.67; 0.17; 0.17	5,48	positive
71102	6	1	10:35	1.02	1.00	-0,96	random
71202	7	1	22:29	0.63	1.00	-2,62	random
71302	4	2	14:54; 01:13	706.72; 0.44	0.50; 0.50	4,51	positive
71402	3	2	00:38; 10:15	692.04; 3.16	0.33; 0.67	2,58	positive
71502	5	3	22:41; 14:29; 03:47	610.16; 612.07; 3.21	0.40; 0.20; 0.40	4,88	positive
71602	11	1	17:20	0.89	1.00	-0,97	random
71702	7	1	17:35	0.81	1.00	-1,83	random
71802	5	2	18:16; 08:37	56.97; 108.86	0.60; 0.40	10,88	very strong
71902	4	2	05:31; 20:12	699.29; 5.75	0.25; 0.75	4,41	positive
72002	3	2	05:41; 13:36	3.78; 598.38	0.67; 0.33	2,9	positive

Table S2 – *Continued from previous page*

Patient ID	N	K	μ (in hours and minutes)	κ	ω	ΔBIC	Evidence categories
72102	4	2	14:36; 21:55	10.36; 226.19	0.50; 0.50	6,28	strong
72202	11	1	01:51	0.27	1.00	-4,41	random
72302	6	3	22:43; 13:21; 02:47	707.54; 8.72; 65.73	0.17; 0.50; 0.33	0,78	weak
72402	10	3	14:25; 22:40; 06:21	5.98; 99.52; 151.14	0.30; 0.30; 0.40	7,1	strong
72502	7	2	03:30; 15:57	2.85; 691.70	0.86; 0.14	2,64	positive
72602	8	3	07:47; 18:38; 00:35	3.85; 91.52; 338.75	0.50; 0.25; 0.25	-1,15	random
72702	8	1	14:34	0.88	1.00	-1,47	random
72802	5	2	03:05; 15:02	5.63; 698.87	0.80; 0.20	5,18	positive
72902	4	3	09:27; 23:33; 15:21	602.32; 600.52; 602.32	0.25; 0.50; 0.25	12,06	very strong
73002	5	1	11:42	0.92	1.00	-1,39	random
75102	6	2	23:09; 09:23	2.48; 623.47	0.83; 0.17	1,08	weak
75202	7	1	22:40	0.68	1.00	-2,42	random
79502	8	2	17:14; 06:37	4.03; 687.77	0.88; 0.12	6,42	strong
80602	5	2	11:30; 06:21	611.48; 7.01	0.20; 0.80	6,04	strong
80702	7	1	09:30	0.73	1.00	-2,18	random
81102	7	2	23:20; 10:39	0.57; 691.47	0.58; 0.42	4,95	positive
81402	7	3	19:30; 06:58; 02:16	698.29; 147.50; 93.96	0.14; 0.29; 0.57	12,46	very strong
81802	8	3	23:05; 06:28; 15:07	189.77; 11.25; 708.15	0.50; 0.25; 0.25	12,32	very strong
85202	7	2	03:48; 16:30	2.64; 113.61	0.72; 0.28	0,68	weak
86202	6	1	05:41	2.45	1.00	4,91	positive
91602	26	1	02:35	1.74	1.00	18,67	very strong
92102	5	3	01:18; 17:59; 07:57	641.94; 641.94; 640.71	0.20; 0.20; 0.60	17,6	very strong
92202	15	1	03:19	0.38	1.00	-4,34	random
93402	5	2	10:22; 00:53	673.53; 2.02	0.20; 0.80	-0,02	random
93902	7	3	09:04; 17:26; 03:17	169.72; 7.96; 25.43	0.43; 0.29; 0.29	1,02	weak
94402	8	1	10:39	0.32	1.00	-3,76	random
95202	7	2	00:31; 14:37	20.48; 95.72	0.71; 0.29	11,84	very strong
95802	8	3	08:44; 16:02; 21:47	701.16; 94.48; 2.03	0.25; 0.36; 0.39	2,78	positive
96002	7	3	08:23; 00:25; 17:58	3.82; 142.57; 27.13	0.29; 0.29; 0.43	-2,68	random
97002	11	2	23:50; 14:21	3.29; 61.07	0.82; 0.18	3,89	positive
98102	5	2	18:50; 05:44	691.17; 11.14	0.20; 0.80	8,13	strong
98202	9	1	07:05	0.51	1.00	-3,29	random
100002	12	1	22:09	0.18	1.00	-4,79	random
101702	5	4	07:36; 20:00; 12:30; 12:30	709.26; 75.71; 709.37; 709.36	0.40; 0.40; 0.20; 0.00	7,26	strong
102202	12	1	16:11	0.56	1.00	-3,18	random
103002	6	2	15:30; 05:24	1.83; 662.39	0.67; 0.33	3,55	positive
103802	7	1	06:14	0.35	1.00	-3,47	random
104602	5	3	06:25; 23:15; 14:03	701.60; 55.31; 6.46	0.20; 0.40; 0.40	-0,13	random
107302	12	1	16:22	0.57	1.00	-3,12	random
107702	8	1	22:01	0.45	1.00	-3,37	random
107802	11	1	21:07	0.23	1.00	-4,5	random
108402	19	1	00:19	0.74	1.00	-1,17	random
109202	5	3	10:01; 03:25; 17:29	685.83; 11.63; 11.95	0.20; 0.40; 0.40	-2,02	random
109502	6	1	09:39	0.89	1.00	-1,5	random
109602	6	3	14:28; 11:52; 10:24	91.82; 705.50; 441.51	0.50; 0.17; 0.33	12,18	very strong
110602	6	3	17:51; 10:07; 21:34	403.16; 10.11; 706.44	0.33; 0.50; 0.17	4,99	positive
111902	6	2	16:30; 07:35	1.83; 400.97	0.67; 0.33	1,29	weak
112402	13	1	06:13	0.23	1.00	-4,78	random
112502	10	1	20:20	0.13	1.00	-4,53	random
112802	6	3	07:47; 16:21; 11:55	27.89; 26.72; 625.32	0.50; 0.33; 0.17	2,43	positive
113902	12	4	11:57; 22:58; 16:55; 05:00	3.26; 93.73; 702.27; 281.75	0.34; 0.25; 0.16; 0.25	0	weak
114602	7	1	01:54	0.62	1.00	-2,64	random
114702	15	1	20:05	0.27	1.00	-4,88	random
114902	6	1	12:28	0.93	1.00	-1,35	random
115002	6	3	08:08; 02:33; 13:20	688.75; 10.48; 688.75	0.17; 0.67; 0.17	4,54	positive
115102	11	1	16:35	0.57	1.00	-3,1	random
115202	9	3	05:42; 00:39; 14:20	13.93; 66.12; 8.25	0.33; 0.44; 0.22	-0,52	random
123902	6	3	17:10; 06:54; 02:14	4.96; 700.25; 57.12	0.33; 0.17; 0.50	1,17	weak
125002	7	1	18:40	0.57	1.00	-2,81	random
132502	11	2	16:37; 07:44	4.94; 12.77	0.45; 0.55	4,49	positive
1233703	3	2	13:58; 08:35	702.98; 676.57	0.33; 0.67	15,4	very strong
1233803	3	2	13:21; 07:10	703.80; 690.15	0.33; 0.67	15,62	very strong
1234303	5	1	09:06	0.90	1.00	-1,45	random

Table S2 – *Continued from previous page*

Patient ID	N	K	μ (in hours and minutes)	κ	ω	ΔBIC	Evidence categories
1235003	4	2	06:14; 12:25	680.84; 4.58	0.25; 0.75	3,62	positive
1235103	6	1	21:32	0.10	1.00	-3,56	random
1235903	7	1	04:59	0.78	1.00	-1,97	random
1236703	20	1	14:36	0.66	1.00	-1,98	random
1241003	5	3	11:21; 06:36; 16:28	705.24; 46.83; 705.24	0.20; 0.60; 0.20	8,44	strong
1242003	7	1	11:36	0.45	1.00	-3,22	random
1242703	5	3	03:20; 16:14; 09:40	708.39; 16.21; 708.37	0.20; 0.60; 0.20	5,2	positive
1243603	5	1	17:18	0.69	1.00	-2,13	random
1244803	6	1	09:37	1.22	1.00	-0,09	random
1245203	8	1	11:42	0.04	1.00	-4,15	random
1256303	3	2	18:58; 09:49	695.29; 14.24	0.33; 0.67	5,98	positive
1259203	5	2	20:02; 02:26	648.53; 15.25	0.20; 0.80	9,37	strong
1270403	6	2	05:47; 13:44	65.35; 3.08	0.50; 0.50	3,05	positive
1272703	5	1	16:16	0.89	1.00	-1,51	random
1299403	3	1	03:26	6.35	1.00	5,59	positive
1300003	4	3	08:45; 22:17; 01:51	11.58; 660.35; 660.35	0.50; 0.25; 0.25	3,76	positive
1300503	4	2	15:28; 08:48	165.74; 707.50	0.50; 0.50	16,06	very strong
1305803	3	2	08:15; 09:33	656.32; 224.82	0.33; 0.67	11,55	very strong
1306003	4	2	02:09; 06:29	14.30; 698.41	0.75; 0.25	7,33	strong
1306203	10	2	07:53; 19:54	0.57; 672.53	0.71; 0.29	0,36	weak
1306903	12	4	10:51; 18:33; 16:33; 08:47	673.12; 671.38; 673.12; 670.46	0.08; 0.50; 0.08; 0.33	37,17	very strong
1307003	3	1	13:05	1.20	1.00	-0,49	random
1307103	8	1	15:03	1.86	1.00	4,24	positive
1307403	6	2	11:10; 15:35	699.34; 11.73	0.17; 0.83	10,3	very strong
1307503	7	1	21:45	0.95	1.00	-1,18	random
1307803	4	2	13:33; 08:19	703.50; 9.82	0.25; 0.75	6,17	strong
1308403	3	2	09:24; 19:13	703.78; 690.16	0.33; 0.67	15,62	very strong
1308503	4	2	06:38; 20:41	3.45; 703.52	0.75; 0.25	2,58	positive
1308603	4	3	09:33; 16:13; 04:01	454.30; 707.94; 707.95	0.50; 0.25; 0.25	10,82	very strong
1308903	8	1	17:09	1.10	1.00	-0,24	random
1309803	5	3	19:01; 03:09; 12:16	94.39; 7.00; 684.28	0.40; 0.40; 0.20	1,07	weak
1310403	4	4	08:57; 23:43; 08:57; 15:25	709.69; 709.69; 709.69; 706.90	0.24; 0.25; 0.01; 0.50	7,76	random
1310803	4	1	06:55	1.04	1.00	-0,97	random
1311003	3	2	09:55; 18:19	689.09; 45.40	0.33; 0.67	8,35	strong
1311203	13	1	21:08	1.00	1.00	0,39	weak
1312603	3	2	05:22; 12:08	3.60; 708.80	0.67; 0.33	2,96	positive
1312703	4	1	00:53	1.75	1.00	1,13	weak
1312803	3	1	03:30	0.54	1.00	-1,79	random
1312903	4	1	04:28	2.57	1.00	3,15	positive
1313003	7	4	11:10; 16:57; 03:31; 22:26	134.10; 703.08; 59.63; 703.08	0.43; 0.14; 0.29; 0.14	4,54	positive
1313403	4	1	01:50	3.43	1.00	4,68	positive
1313903	4	3	15:46; 21:52; 10:12	81.39; 661.30; 661.30	0.50; 0.25; 0.25	7,7	strong
1314103	4	3	16:30; 09:52; 20:02	688.17; 18.79; 688.17	0.25; 0.50; 0.25	4,8	positive
1314703	3	2	20:07; 07:11	684.11; 2.53	0.33; 0.67	1,99	weak
1314803	7	1	16:44	1.34	1.00	0,8	weak
1315003	4	3	16:37; 22:57; 05:08	10.30; 626.42; 626.42	0.50; 0.25; 0.25	3,41	positive
1315203	4	2	21:34; 07:54	3.87; 692.30	0.75; 0.25	3	positive
1315403	4	2	09:10; 22:39	5.29; 692.77	0.75; 0.25	4,12	positive
1316003	3	1	00:13	4.41	1.00	4,33	positive
1316303	3	2	04:35; 09:54	678.20; 7.84	0.33; 0.67	4,7	positive
1316403	7	3	14:29; 10:12; 20:25	671.76; 12.25; 624.09	0.14; 0.57; 0.29	7,2	strong
1316503	4	2	03:32; 18:21	38.29; 43.79	0.50; 0.50	5,67	positive
1317003	3	1	08:01	105.06	1.00	14,27	very strong
1317203	3	2	15:32; 20:40	8.43; 606.29	0.67; 0.33	4,76	positive
1317303	4	3	08:32; 03:54; 14:15	707.49; 707.50; 23.24	0.25; 0.25; 0.50	5,31	positive
1317403	3	2	16:05; 10:55	32.71; 709.29	0.67; 0.33	7,71	strong
1317903	4	2	01:24; 11:47	5.51; 708.12	0.75; 0.25	4,28	positive
1318803	3	1	14:43	1.18	1.00	-0,55	random
1319103	4	1	16:12	0.74	1.00	-1,79	random
1319203	7	1	21:48	0.45	1.00	-3,2	random
1320303	3	2	12:03; 17:36	687.33; 77.57	0.33; 0.67	9,24	strong
1320503	3	2	04:02; 18:12	700.68; 6.98	0.33; 0.67	4,48	positive
1320603	3	2	09:03; 15:13	613.35; 4.25	0.33; 0.67	3,22	positive

Table S2 – *Continued from previous page*

Patient ID	N	K	μ (in hours and minutes)	κ	ω	ΔBIC	Evidence categories
1320903	4	1	07:03	0.67	1.00	-1,94	random
1321003	3	2	10:35; 21:10	684.22; 3.21	0.33; 0.67	2,62	positive
1321803	5	3	17:08; 22:26; 05:47	680.03; 167.80; 13.23	0.20; 0.40; 0.40	3,31	positive
1321903	5	3	22:54; 04:52; 15:10	708.67; 708.68; 20.38	0.20; 0.20; 0.60	5,9	positive
1322103	11	2	11:38; 03:46	2.86; 85.26	0.55; 0.45	9,23	strong
1322703	3	1	22:24	2.02	1.00	1,26	weak
1322803	4	2	18:40; 12:49	5.87; 705.06	0.75; 0.25	4,5	positive
1322903	4	3	12:33; 16:05; 08:37	655.51; 655.51; 111.42	0.25; 0.25; 0.50	8,43	strong
1323803	5	4	21:00; 09:45; 04:06; 04:06	29.88; 702.21; 702.21; 702.21	0.60; 0.20; 0.09; 0.11	2,23	positive
1324103	4	2	17:22; 11:20	19.68; 669.58	0.75; 0.25	8,32	strong
1324503	11	1	01:01	0.56	1.00	-3,18	random
1324803	9	3	17:50; 03:41; 06:22	4.28; 271.66; 709.65	0.56; 0.33; 0.11	3,91	positive
1324903	10	1	14:40	1.13	1.00	0,58	weak
1325103	4	2	06:31; 00:26	12.06; 700.95	0.75; 0.25	6,81	strong
1325403	4	3	21:47; 12:54; 19:06	692.17; 16.89; 692.17	0.25; 0.50; 0.25	4,61	positive
1325603	4	2	07:00; 20:40	3.52; 10.59	0.50; 0.50	-2,34	random
1325903	5	3	03:29; 11:59; 07:14	18.22; 693.62; 188.46	0.40; 0.20; 0.40	4,33	positive
1326003	7	3	05:57; 14:33; 01:37	3.70; 659.01; 640.63	0.58; 0.14; 0.28	3,72	positive
1326103	4	2	20:10; 07:27	686.94; 73.22	0.25; 0.75	12,31	very strong
1326503	3	2	14:57; 10:12	10.57; 690.62	0.67; 0.33	5,38	positive
1327403	7	1	00:39	0.42	1.00	-3,3	random
1327603	7	1	16:43	1.17	1.00	-0,08	random
1327903	8	1	02:31	3.88	1.00	12	very strong
1328403	4	3	08:12; 17:06; 23:48	3.80; 705.28; 705.28	0.50; 0.25; 0.25	1,38	weak
1328603	8	1	01:28	0.21	1.00	-3,98	random
1328803	5	1	18:01	1.46	1.00	0,6	weak
1328903	5	3	04:42; 11:20; 23:04	15.46; 252.00; 708.52	0.40; 0.40; 0.20	4,94	positive
1329003	9	3	14:55; 05:56; 22:55	31.61; 13.09; 620.67	0.56; 0.33; 0.11	5,32	positive
1329303	3	2	17:52; 08:45	686.20; 2.47	0.33; 0.67	1,93	weak
1329503	4	2	23:31; 04:41	694.47; 39.85	0.25; 0.75	10,48	very strong
1330103	3	3	15:55; 15:55; 05:36	704.39; 704.40; 163.59	0.01; 0.33; 0.67	7,66	random
1330203	4	3	01:33; 06:12; 20:58	83.64; 679.88; 679.89	0.50; 0.25; 0.25	7,81	strong
1330903	9	2	08:37; 02:20	41.42; 3.86	0.43; 0.57	5,47	positive

BIC stands for Bayesian Information Criterion, K represents the number of clusters/components, N the number of lead seizures, and μ, κ, and ω for the model parameters mean direction, concentration and component weight, respectively

References

1. Klatt J, Feldwisch-Drentrup H, Ihle M, Navarro V, Neufang M, Teixeira C, et al. The EPILEPSIAE database: An extensive electroencephalography database of epilepsy patients. Epilepsia. 2012 9;53(9):1669-76.
2. Konschelle F. Mixture of von Mises Distribution; 2019. Available from: https://framagit.org/fraschelle/mixture-of-von-mises-distributions.
